# Diagnostic sensitivity and specificity of metagenomic sequencing and qPCR for detection of viruses associated with bovine respiratory disease estimated using Bayesian latent class models

**DOI:** 10.3389/fvets.2026.1704414

**Published:** 2026-02-25

**Authors:** Emmanuel Donbraye, Lianne McLeod, Zhijian Chai, Stacey R. Lacoste, E. Luke McCarthy, Janet E. Hill, Nathan E. N. Erickson, Matthew G. Links, Simon J. G. Otto, Yanyun Huang, Cheryl L. Waldner

**Affiliations:** 1 Department of Large Animal Clinical Sciences, Western College of Veterinary Medicine, University of Saskatchewan, Saskatoon, SK, Canada; 2 Department of Animal and Poultry Science, College of Agriculture and Bioresources, University of Saskatchewan, Saskatoon, SK, Canada; 3 Department of Veterinary Microbiology, Western College of Veterinary Medicine, University of Saskatchewan, Saskatoon, SK, Canada; 4 HEAT-AMR (Human-Environment-Animal Transdisciplinary AMR) Research Group, School of Public Health, University of Alberta, Edmonton, AB, Canada; 5 Centre for Healthy Communities, School of Public Health, University of Alberta, Edmonton, AB, Canada; 6 Prairie Diagnostic Services Inc., Saskatoon, SK, Canada; 7 Department of Veterinary Pathology, Western College of Veterinary Medicine, University of Saskatchewan, Saskatoon, SK, Canada

**Keywords:** Bayesian latent class models, bovine respiratory bacteria, bovine respiratory disease, bovine respiratory viruses, long-read metagenomic sequencing, qPCR

## Abstract

**Introduction:**

Very few studies have examined the diagnostic sensitivity and specificity of currently available laboratory tests for detecting respiratory pathogens in cattle, and even fewer have examined test performance on samples from animals before the onset of clinical disease.

**Methods:**

In this study, Bayesian latent class modeling (BLCM) was used to assess diagnostic test performance in the absence of a gold standard on nasal swabs collected from 19 western Canadian feedlots. Viruses associated with bovine respiratory disease (BRD) were identified using qPCR from a commercial diagnostic laboratory from 760 nasal swabs collected from fall-placed calves (FPC) and yearlings (YRL) at and shortly after feedlot arrival. Using BLCM, the qPCR results were compared to previously reported matching nanopore metagenomic sequencing data for these same samples. Based on BLCM, test sensitivities and specificities were estimated for the detection of bovine coronavirus (BCoV), bovine herpesvirus type 1 (BoHV-1), bovine parainfluenza virus type 3 (BPIV-3), bovine respiratory syncytial virus (BRSV), and influenza D virus (IDV). Estimates informed by BLCM were not available for the detection of bovine viral diarrheal virus (BVDV) because qPCR did not detect this virus in any samples.

**Results:**

Diagnostic sensitivity of qPCR was higher than metagenomic sequencing for detecting BCoV (qPCR 0.90, 95% CrI 0.81–0.99; sequencing 0.35, 95% CrI 0.25–0.46) and BoHV–1 (qPCR 0.39, 95% CrI 0.19–0.99; sequencing 0.04, 95% CrI 0.01–0.15). However, the estimated diagnostic sensitivity of metagenomic sequencing was higher than qPCR for identifying BRSV (qPCR 0.32, 95% CrI 0.22–0.43; sequencing 0.60, 95% CrI 0.44–0.77). No significant difference among sensitivities was noted for the detection of BPIV-3 (qPCR 0.42, 95% CrI 0.21–0.66; sequencing 0.52, 95% CrI 0.19–0.87) and IDV (qPCR 0.65, 95% CrI 0.53–0.79; sequencing 0.60, 95% CrI 0.48–0.73). Diagnostic specificity was comparable for most viruses, except for BCoV, where metagenomic sequencing (BCoV 0.91, 95% CrI 0.88–0.95) outperformed qPCR (BCoV 0.59, 95% CrI 0.51–0.68). The specificity and sensitivity for detection of BRD-associated bacteria from the same metagenomic data were also similar to those estimated for culture and qPCR results for the same samples.

**Discussion:**

Estimated test sensitivities of both nanopore metagenomic sequencing and qPCR for the detection of BRD viruses of interest in nasal swab samples were moderate to very low for most viruses. While the tests varied in their ability to detect individual viruses, data from this study suggest nanopore metagenomic sequencing offers a potential alternative for diagnostic laboratories to identify three of six important BRD viruses as well as bacteria associated with BRD.

## 1 Introduction

Bovine respiratory disease (BRD) in feedlot cattle can be the direct result of viral damage to the respiratory epithelium or the indirect result of compromised immune function, which in turn increases the risk of secondary bacterial infections and pneumonia in calves (1, 2). Several viruses have been consistently associated with BRD, including bovine herpesvirus type 1 (BoHV-1; *Bovine alphaherpesvirus* 1) (3), bovine respiratory syncytial virus (BRSV; *Bovine orthopneumovirus*) (4), bovine viral diarrhea virus (BVDV-1, *Pestivirus bovis* and BVDV-2, *Pestivirus tauri*) (5), and bovine parainfluenza virus type 3 (BPIV-3; *Bovine respirovirus-*3) (6). More recently, bovine coronavirus (BCoV; *Betacoronavirus 1*) (7, 8) and influenza D virus (IDV; *Deltainfluenzavirus influenzae*) (8, 9) have also been associated with BRD.

Conventional diagnostic methods for the detection of BRD-associated viruses have important limitations. Polymerase chain reaction (PCR) and its derivatives, including multiplex PCR, real-time PCR, and reverse transcriptase real-time PCR (rt-PCR) assays, are typically considered the gold standards for diagnosing viruses associated with BRD. Quantitative real-time PCR (qPCR) is a molecular technique that amplifies and simultaneously quantifies a targeted DNA molecule. It uses fluorescence to monitor the accumulation of PCR products in real time, allowing for both the detection and quantification of nucleic acids within a sample (10). PCR methods are limited to detecting viruses that have been comprehensively characterized and for which appropriate primers and probes have been developed (11). These very targeted diagnostic methods are limited in their ability to detect viruses not included in the testing panel. Furthermore, evolving viral genomes that no longer match existing PCR primers and probes may go undetected during identification (12). Furthermore, the number of targets that can be simultaneously tested using PCR-based assays is limited by instrumentation and incompatibility of fluorophores (13). In multiplex qPCR, each target is detected using a unique fluorophore with distinct excitation and emission spectra (14). However, overlapping spectral properties of these dyes can lead to signal crosstalk between detection channels, compromising the accuracy of the assay. Spectral overlap limits the number of fluorophores—and thus targets—that can be reliably distinguished in a single reaction (15).

Metagenomic sequencing allows the identification of a subset of nucleic acids in a sample and does not require specific *a priori* targets (16). This makes it a promising option for diagnostics, especially for RNA viruses that are less stable than DNA viruses. RNA molecules are susceptible to degradation due to their single-stranded structure and vulnerability to temperature, pH, and enzymatic activity. These factors can compromise diagnostic assays, leading to false-negative results (17). The high mutation rates of RNA viruses present challenges for molecular diagnostics, as changes in viral genomes can reduce detection sensitivity (13, 18). This ability to identify a wider range of possible pathogens in a sample offers important potential for infectious disease diagnostics (19). Metagenomic sequencing has been used to detect viruses in cattle with BRD without the need for the development of virus-specific assays (8, 9, 20–22).

Metagenomic studies successfully reported increasing numbers and identity of viruses present in clinical BRD samples, as well as associations between lesser-known viruses and BRD. For instance, using metagenomic sequencing Ng and colleagues (9) detected nine viruses in nasal samples from young dairy calves and found significant associations between BRD and the detection of bovine adenovirus 3 (BAdV3), bovine rhinitis A virus (BRAV), and IDV (9). In two other metagenomic studies in feedlot cattle, in which each identified 21 viruses, bovine rhinitis B virus (BRBV), BRAV, and IDV were associated with BRD (8, 22).

Diagnostic sensitivity (Se) and specificity (Sp) can be used as measures of diagnostic test performance, indicating the ability of a test to correctly identify individuals with and without a particular condition or pathogen; such information is crucial for diagnosis and treatment planning (23). Researchers typically assess the efficacy of a novel diagnostic test by comparing performance relative to a reference test that is intended to reflect the true status of the individual (24). Philosophically, the reference test is assumed infallible or a “gold standard,” however the reference test itself can never be perfect. Most traditional studies measure the “apparent” performance relative to the reference test, rather than the actual performance (25).

Bayesian latent class models estimate the accuracy of diagnostic tests without the assumption of a gold standard reference test with 100% Se and Sp. These models allow the inclusion of true disease status as a latent variable and then use a cross-classification of outcomes from two or more diagnostic tests applied to two or more populations (between which prevalence is expected to vary) to estimate diagnostic Se and Sp of each of the tests (26, 27).

In veterinary medicine, Bayesian latent class models (BLCMs) have been used to assess diagnostic tests for emerging and re-emerging pathogens, estimate true disease prevalence, manage complex data structures, assess pooled testing, and demonstrate disease absence (28). The models have been employed in studies of numerous animal diseases, such as foot and mouth disease in cattle (29), African swine fever in pigs and boars (30), highly pathogenic avian influenza in poultry (31), paratuberculosis in dairy goats, sheep, and cattle herds (32, 33), and both bovine tuberculosis (34) and brucellosis (35) in cattle herds, among others.

To date, few studies have evaluated the effectiveness of metagenomic sequencing relative to diagnostic tests for identifying bovine respiratory viruses or bacteria. For example, Zhang and colleagues (36) conducted a traditional assessment comparing nanopore sequencing to qPCR for detecting IDV and then using qPCR as a reference test for the assessment of Illumina-based metagenomic sequencing. In another study, BLCM was used to evaluate the detection of *Mycoplasmopsis bovis* using nanopore metagenomic sequencing, triplex qPCR assay, and matrix-assisted laser desorption ionization-time of flight (MALDI-TOF) mass spectrometry (37). To the best of our knowledge, no study has employed BLCMs to compare the clinical sensitivities and specificities of metagenomic sequencing and qPCR in the detection of BRD viruses without assuming qPCR is a gold standard reference test.

Veterinary practitioners in North America currently have limited diagnostic options for BRD virus detection, with qPCR being the only widely available test. Evaluating how a metagenomic protocol performs under real-world field conditions provides critical insight into its practical utility and potential as a complementary or alternative diagnostic tool. Therefore, the primary objective of this investigation was to use BLCMs to estimate the diagnostic test performance (Se and Sp) of both nanopore metagenomic sequencing and qPCR from a diagnostic laboratory for the detection of viruses in nasal swabs obtained from cattle in commercial feedlots in western Canada at arrival processing and again approximately 2 weeks later. The secondary objective of this study was to use BLCMs to estimate the performance of this metagenomic protocol optimized for the detection and characterization of viruses when concurrently detecting four respiratory bacteria of interest.

## 2 Materials and methods

### 2.1 Ethics statement

This study was conducted following the recommendations of the Canadian Council of Animal Care (CCAC) (38). The Animal Care and Use Committee at the University of Alberta (ACUC Livestock—University of Alberta AUP00004110) approved the ethics protocol and standard operating procedure for nasal swab collection, which was shared with the Research Ethics Board at the University of Saskatchewan (USask AREB File Number 20220072) and the Animal Care Committee of Feedlot Health Management Services as a study partner.

### 2.2 Study population and sampling procedure

As described in the companion study (21), data for the current study were generated in partnership with the Canadian Integrated Program for Antimicrobial Resistance Surveillance (CIPARS) as part of a nationwide, longitudinal survey of antimicrobial resistance in feedlot cattle. CIPARS collaborated with veterinary practices that identified volunteer feedlot operators representing various feedlot sizes and geographic regions across the Canadian feedlot industry (39). In addition to routine collection by CIPARS of deep nasopharyngeal swabs for culture and susceptibility testing, nasal swabs were also collected in the fall of 2022 for viral nanopore metagenomic sequencing (additional methods and descriptive data reported in the companion paper (21)) and qPCR analysis (methods and data reported in this paper).

Privately owned veterinary clinics collected nasal swabs from fall-placed calves (FPC) and yearlings (YRL) arriving at 19 commercial feedlots in Alberta between September and December 2022. This sampling occurred during the “fall run,” when FPC are at highest risk of developing BRD (40, 41).

Skilled registered veterinary technologists used commercial chutes and headgates with neck extenders to restrain cattle for swab collection. They obtained a single short nasal swab from a convenience sample of 20 cattle from each of 13 pens of FPC (*n* = 260) and six pens of YRL (*n* = 120) during initial arrival processing before metaphylaxis and that would be used for both qPCR and nanopore metagenomic sequencing. One pen was sampled per feedlot. A second set of convenience samples from 20 cattle in the same pens (*n* = 260 + 120) were taken again at approximately 14 days of feed (DOF), without intentionally resampling the same individual cattle. Approximately 3 cm of the swab tip was cut and placed in 1 mL of liquid Amies transport media (CoPan Diagnostics, Carlsbad, CA, United States) for transportation on ice packs in coolers to the Prairie Diagnostic Services Inc. (PDS) laboratory at the University of Saskatchewan (Saskatoon, SK, Canada). Samples were delivered cold, not frozen, and stored at −80 °C until processing.

### 2.3 Host depletion, nucleic acid extraction, and purification

Nucleic acid extraction is described in the companion paper (21). In brief, samples in tubes were briefly thawed in a room-temperature water bath and then stored on ice during processing. Each of the samples and extraction negative controls (molecular biology grade water for each batch) underwent centrifugation at 13,000 × *g* for 5 min to pellet cellular and non-cellular debris and then collect the supernatant for subsequent analysis. To degrade host extracellular nucleic acids during the host depletion stage, 500 μL of the clarified sample supernatant were mixed with 34 μL of nuclease digestion master mix (six units of TURBO DNase, 20 μL of TURBO DNase buffer, and 20 units of RNase I from Invitrogen, Waltham, MA, United States) and incubated at 37 °C for 90 min. This was followed by the purification of viral nucleic acids using the QIAamp MinElute Virus Spin Kit (QIAGEN, Hilden, Germany), following the manufacturers’ instructions. Briefly, carrier RNA was added to the mixture, then proteins were digested with protease contained in the kit at 56 °C for 15 min and then treated with 95% ethanol to halt enzyme activity. The lysates were incubated for 5 min at room temperature before being applied to MinElute columns (QIAGEN, Hilden, Germany) on vacuum manifolds. The lysate passed through the purification column, and the bound sample, containing the nucleic acids, was washed and treated with 95% ethanol in the column. After washing to remove contaminants, the samples were spun and dried at 56 °C for 3 min to dry the membrane further. Subsequently, the sample nucleic acid bound to the column were eluted in 40 μL of buffer AVE by incubation for 5 min at room temperature and spinning at 17,000 × *g* for 1 min. The eluted nucleic acid was then divided into aliquots, with one aliquot used for viral metagenomics and the other for qPCR analysis.

### 2.4 qPCR analysis for respiratory viruses

Quantitative reverse transcriptase PCR (qPCR) was completed by the regional diagnostic laboratory (PDS, Saskatoon, SK, Canada). The AgPath-ID One-Step RT-PCR Kit (Applied Biosystems, Thermo Fisher Scientific, Waltham, MA, United States) was used with previously described specific primers and probes (Table 1) including for bovine 18S rRNA gene (internal amplification control, IAC) (42), BCoV (43), BPIV-3 (44), BRSV (45), IDV (46), and BVDV [VetMAX™-Gold BVDV PI Detection Kit (Applied Biosystems, Waltham, MA, United States)]. BCoV, BRSV, and IDV reactions were completed as a triplex. BPIV-3 and BDVD were multiplexed in duplexes with the bovine 18S rRNA gene assay. Each reaction contained a final concentration of 1 × RT-PCR enzyme mix, 12.5 μL 2 × RT-PCR buffer, forward and reverse primers at a final concentration of 10 μM, and 5 μL purified nucleic acids. The bovine 18S rRNA gene probe was added to a final concentration of 1 μM. All other probes were added to a final concentration of 5 μM. Where necessary, water was added to bring the reactions to 25 μL final volume. Samples were incubated at 48 °C for 30 min, then polymerase enzyme activation was performed at 95 °C for 10 min. Amplification occurred for 40 cycles at 95 °C for 15 s, then 60 °C for 60 s.

**Table 1:** Primer and probe sets used for qPCR analysis.

Target	Target gene	F/R/P	Sequence 5′–3′	Fluorophore/quencher	References
BCoV*	M gene	F	CTGGAAGTTGGTGGAGTT	FAM/MGB-NFQ	(43)
		R	ATTATCGGCCTAACATACATC		
		P	CCTTCATATCTATACACATCAAGTTGTT		
BoHV-1*	gb gene	F	TGTGGACCTAAACCTCACGGT	FAM/3′-TAMRA (FRET-Based Quencher)	(47)
		R	AGGACCGCGAGTTCTTGCCGC		
		P	GTAGTCGAGCAGACCCGTGTC		
BPIV-3*	Mprot	F	TGTCTTCCACTAGATAGAGGGATAAAATT	FAM/MGB-NFQ	(44)
		R	GCAATGATAACAATGCCATGGA		
		P	ACAGCAATTGGATCAATAA		
BRSV*	F gene	F	AAGGGTCAAACATCTGCTTAACTAG	Cy5/BHQ1	(45)
		R	TCTGCCTGWGGGAAAAAAG		
		P	AGAGCCTGCATTRTCACAATACCACCCA		
BVDV	5′ UTR		**	FAM/	VetMAX™-Gold Applied Biosystems™ 4,413,938
IDV*	PB1	F	TGGATGGAGAGTGCTGCTTC	VIC/HEX	(46)
		R	GCCAATGCTTCCTCCCTGTA		
		P	CATGTTAAACATTCCCATCAGCATTCCT		
18S	18S rRNA gene	F	GATTAAGTCCCTGCCCTTT	Cy5/BHQ2	(42)
		R	GATAGTCAAGTTCGACCGTCTT		
		P	CACACCGCCCGTCGCTACTACC		

F, forward primer; R, reverse primer; P, probe; BRSV, bovine respiratory syncytial virus; BPIV-3, bovine parainfluenza virus 3; BCoV, bovine coronavirus; IDV, influenza D virus, BoHV-1, bovine herpesvirus 1; 18S, bovine 18S rRNA gene. * Information on in-house analytical validation of qPCR method adaptations was reported in Supplementary material 1. ** Details are held as proprietary information.

BoHV-1 qPCR (47) was performed as a duplex TaqMan assay along-side bovine 18S rRNA using 5 μL purified nucleic acids (Applied Biosystems, Thermo Fisher Scientific, Waltham, MA, United States) (Table 1). TaqMan Fast Advanced Master Mix was diluted to 1 × and combined with forward and reverse primers to a final concentration of 10 μM. The probe was added to a final concentration of 5 μM for BoHV-1 and 1 μM for the bovine 18S rRNA gene probe. Reactions were topped up with water to a final volume of 18 μL. Enzymatic activation of the reaction mix occurred at one cycle of 95 °C for 2 min, and amplification occurred at 40 cycles at 95 °C for 5 s, followed by 60 °C for 33 s.

All qPCR reactions were conducted using one of the following thermal cyclers: the Bio-Rad CFX96 (Bio-Rad Laboratories, Hercules, CA, United States), Bio-Rad Opus96 (Bio-Rad Laboratories, Hercules, CA, United States), or ABI7500 Fast (ABI/Thermo Fisher Scientific, Waltham, MA, United States). Data acquisition and analysis were performed using BioRad Maestro 2.3 software v.5.3.022.1030 (Bio-Rad Laboratories, Hercules, CA, United States).

All samples were evaluated in duplicate alongside the positive controls (internally generated or commercially acquired) for each virus, no template controls, and negative controls (commercially acquired with positive controls). Samples were considered positive for a specific virus if the IAC and both test duplicates gave a sigmoid amplification curve with a Ct (cycle threshold) value of ≤ 38.0. The source, targets, and results for the positive controls are described in Supplementary Table S1.1.

The validation work reported by the commercial laboratory as part of adapting previously reported primers and probes for the assays described above was summarized in Supplementary material 1, along with parameters used for an *in silico* assessment of the suitability of the multiplexed assays. Assay results for the reported qPCRs. In short, we used thermonucleotideBLAST (48) and primersearch (49) to identify any possible putative off-target amplicons using the *B. taurus* and viral genomes from RefSeq. The only possible off-target amplicons we identified were all in excess of 18 Mbps, providing no evidence to concern the commercial laboratory’s use of the multiplex assays.

### 2.5 Nanopore sample preparation, library preparation, and sequencing

Sample preparation, library preparation, and sequencing are described elsewhere (21). As previously described for reverse transcription using SuperScript IV First-Strand Synthesis Kit (Applied Biosystems, Waltham, MA, United States), 10 μL of extracted nucleic acids from each sample were combined with 3.5 μL of a random primer mastermix containing 1 μM FR26RV-N primer (5′-GCC GGA GCT CTG CAG ATA TCN NNN NN-3′), 0.5 mM dNTPs, and 0.42 μL of DEPC-treated water. The mixture was subsequently incubated at 65 °C for 5 min to facilitate the annealing of the random primer to the template (50). After incubation, each sample was placed on ice for 1 min and then combined with 7 μL of reverse transcription master mix (4 μL Superscript IV 5X buffer, 1 μL DTT, 1 μL RNase inhibitor, and 1 μL RTase; Invitrogen, Waltham, MA, United States). The mixture was then incubated at 23, 55, and 80 °C, each for 10 min. Following incubation, samples were placed on ice for 1 min before adding 1 μL of *Escherichia coli* RNase H (5 U/μL) to each reaction, which were then incubated at 37 °C for 20 min to terminate the reaction and degrade the RNA.

Second-strand synthesis was performed using Sequenase DNA Polymerase (Applied Biosystems, Waltham, MA, United States) according to manufacturers’ instructions. Samples were allowed to cool to room temperature for 10 min and 10 μL of Sequenase mastermix was added, which comprised 2 μL 5X Sequenase buffer, 0.3 μL Sequenase enzyme, and 7.7 μL RNase-free water (Applied Biosystems, Waltham, MA, United States). The mixture was then incubated with the following cycling conditions: a slow ramp from 10 to 37 °C over 8 min, maintaining a temperature of 37 °C for 8 min, increasing to 94 °C for 2 min, and finally 10 °C for 5 min. Following this, an additional 0.3 μL of Sequenase enzyme and 0.9 μL of dilution buffer were added, and the reaction incubated under the same conditions.

After conducting second-strand synthesis, each sample and extraction negative control was purified and size-selected with a 1X AMPure XP bead suspension (Beckman Coulter, Brea, CA, United States) according to the manufacturer’s instructions, resulting in a final elution volume of 10 μL. Eight μL of purified dsDNA were then combined with 42 μL of amplification mastermix (comprising 1X Standard Buffer, 100 μM dNTP, 1.5 mM MgCl_2_, 0.5 μM FR20RV primer, 0.25 μL polymerase; NEB, Ipswich, MA, United States), and the mixture cycled through the following reaction conditions: 10 min of denaturation at 94 °C, followed by 40 cycles of 94 °C for 1 min, 65 °C for 1 min, and 72 °C for 3 min, and finally a 5-min extension at 72 °C.

The amplified DNA from each sample was purified and selected for size using 0.4x AMPure XP beads (Beckman Coulter, Brea, CA, United States) and normalized to 50 ng before library preparation. Library preparation followed the protocol for Oxford Nanopore Technology’s “Ligation sequencing gDNA—Native Barcoding kit 96 V14” (SQK-NBD114.96, Oxford Nanopore Technologies, Oxford, United Kingdom) in a 96 well plate high-throughput library format with modifications to minimize potential misclassification of barcoded samples or background barcode crosstalk (51, 52). This process involved barcode ligation, adding 1 μL of EDTA (Invitrogen, Waltham, MA, United States), a 10-min room temperature incubation, and a 10-min 65 °C incubation. Barcoded DNA samples were then pooled in groups of 20 from the same feedlot pen and three library preparation water (negative) controls per library to detect and account for any potential contamination that may have occurred during the library preparation or sequencing process (53). Following library preparation, samples and water controls were submitted as a group for sequencing on the same flow cell. Extraction negative controls were submitted as a group on a separate flow cell with additional library preparation water controls.

Twenty ng of each prepared library were delivered to the Omics and Precision Agriculture Laboratory (OPAL, University of Saskatchewan) for Oxford Nanopore Technologies (ONT) sequencing using the PromethION24 platform. The ONT R10.4.1 flow cells (Oxford Nanopore Technologies, Oxford, United Kingdom) were loaded according to standard procedures and sequencing was performed for 48 h with default run parameters using high-accuracy Guppy real-time base calling with a Q-score cutoff of 9. The MinKNOW platform QC check confirmed that more than 5,000 pores were available for each flow cell.

### 2.6 Bioinformatics analysis

Bioinformatics analysis was described in the companion paper (21). Fastq_pass folder data from MinKNOW were processed using Porechop v0.2.4 (54) to remove primers or adapters, with default parameters applied. NanoFilt (version 2.8.0) was used to discard reads shorter than 200 bps, while Nanostat (version 1.6.0) provided statistics about the distribution of read length by total base pairs per sample (55).

Kraken2 (version 2.1.2) was used to perform taxonomic classification of reads, with a minimum confidence of 0.05 (56). A customized database was utilized for Kraken 2 classification, which incorporated bacterial, viral, and archaeal subsets of the November 2023 RefSeq database (57), as well as the *Bos taurus* ARS-UCD1.2_Btau5.0.1Y genome assembly (58, 59). Ordinarily, sequences identified as host are eliminated prior to subsequent analysis; however, we consistently observed a minor fraction of hybrid *B. taurus* bacterial reads (less than 0.1% of total reads) in the samples. A bespoke software, kmer_filter.py, was developed to recover host-classified reads that satisfied a threshold of 25% non-host sequence using Kraken2 k-mer identification and incorporate these reads for further analysis. Non-host reads (i.e., those not categorized under the *B. taurus* taxid 9,913) were extracted using the KrakenTools v1.2 (60) utility extract_kraken_reads.py, and these were combined with the hybrid reads using a combination of bash utilities and the BBTools v38.86 (61) filterbyname.sh script. To enhance the species-level abundance estimation reported by Kraken 2, Bracken v2.7 (62) was employed with a minimum read length of 200 bp (“--read-length 200”). Reads classified as host were excluded from further analysis. A custom script, report_taxon_read_lengths.py, added additional context to the Bracken results, including the total amount of sequence in base pairs reported for each species (including child taxa) and the fraction of total classified sequence. Further details and all custom scripts can be found at https://github.com/coadunate/ASSETS_2.

### 2.7 Statistical analysis

Descriptive statistical methods were used to characterize the qPCR results. Initial statistical analyses for nanopore metagenomic sequencing are described in the companion paper (21). Data summaries included an overall read count for each virus and the proportion of samples exhibiting at least one detected read for each virus. Before statistical analysis, the mean read count of the water controls for each virus for each flow cell was subtracted from the observed read count of the corresponding virus in each sample for the corresponding flow cell. This adjustment was implemented to address potential misclassification issues among barcoded samples or barcode crosstalk (51, 52).

### 2.8 Bayesian latent class analyses for virus assays

**Figure 1:**
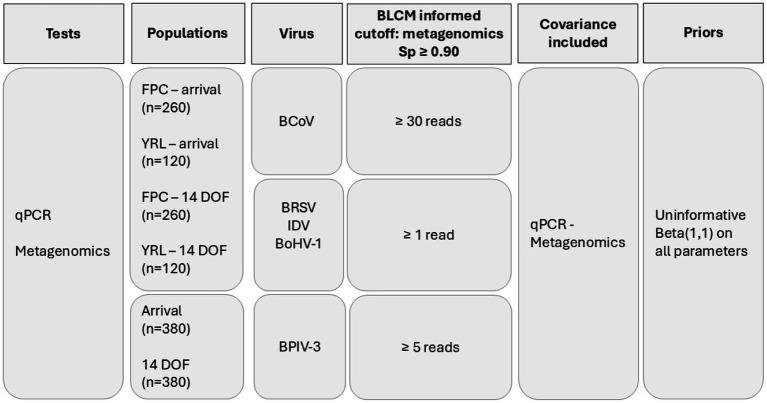
Summary of the methods for the final Bayesian latent class models comparing metagenomics and qPCR reported for five respiratory viruses detected in nasal swabs across four populations of feedlot cattle. BCoV, bovine coronavirus; BoHV-1, bovine herpes virus; BRSV, bovine respiratory syncytial virus; BPIV-3, bovine parainfluenza virus 3; BVDV, bovine viral diarrhea virus 1 and 2; IDV, influenza D virus; BLCM, Bayesian latent class model; qPCR, quantitative polymerase chain reaction; FPC, fall placed calves; YRL, yearlings; DOF, days on feed.

BLCMs were developed to estimate the Se and Sp of the viral metagenomic sequencing protocol and a commercial qPCR for the detection of six viruses associated with BRD. BVDV-1 and BVDV-2 were combined as the qPCR used did not discriminate between types. The number of metagenomic sequencing reads for BCoV, BoHV-1, BPIV-3, BRSV, BVDV, and IDV were used to classify samples as positive or negative for each virus. A series of BLCMs were developed to determine an optimal number of reads to use as a positive cutoff for each virus (Figure 1).

**Figure 2:**
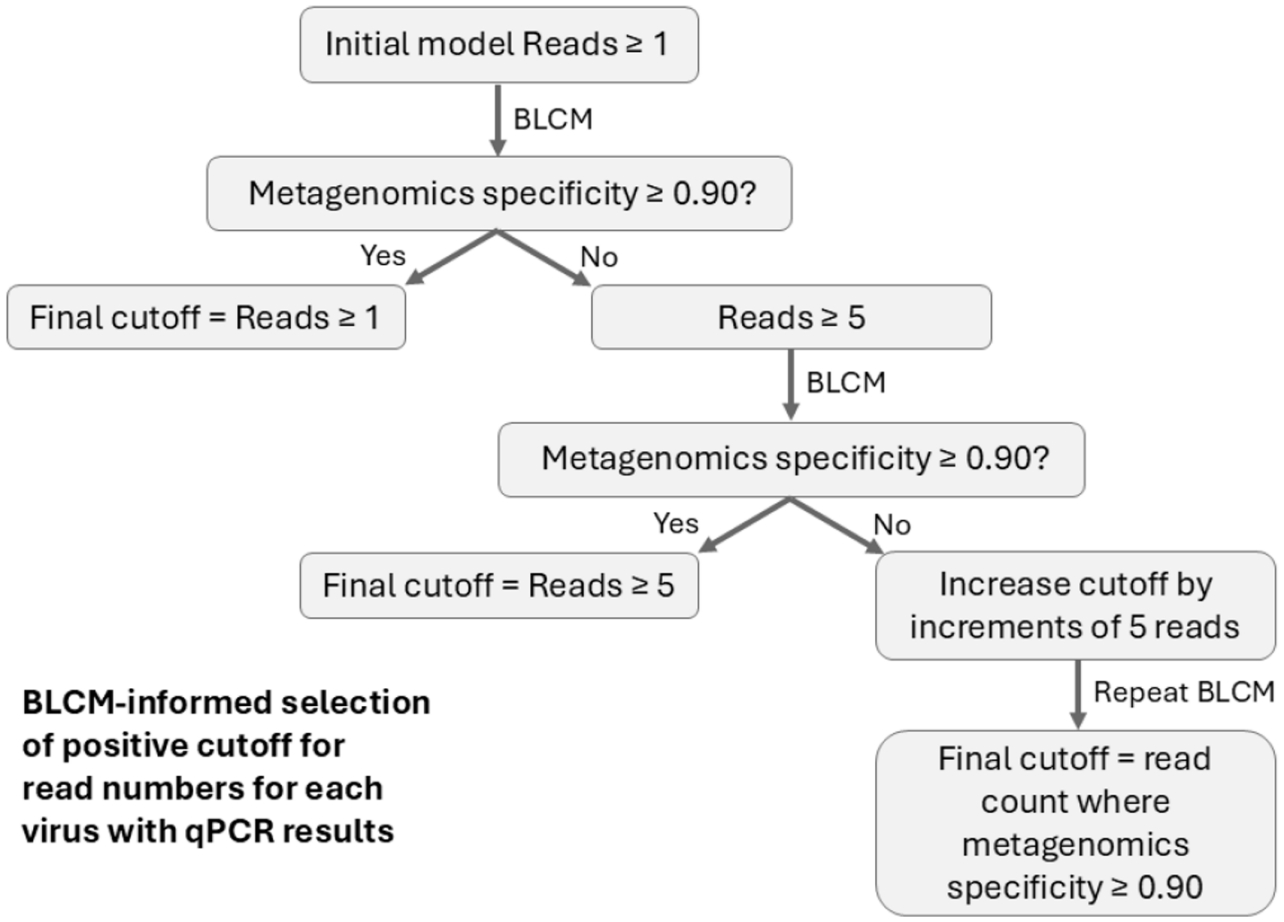
Summary of the workflow for determining thresholds for identifying samples containing target viruses based on viral read counts as informed by Bayesian latent class models. BLCM, Bayesian latent class model; qPCR, quantitative polymerase chain reaction. The corresponding figure summarizing the workflow for establishing read count cutoffs for classifying bacteria is reported in Supplementary Figure S3.1.

A Sp threshold of ≥0.90 from the BLCM for metagenomic testing was selected to determine the minimum read cutoff for each virus (Figure 2). Given the observed distribution of read counts, detection of ≥1 read was used to define a positive sample for the initial metagenomics model for each virus. For viruses where classification as positive based on ≥1 read resulted in a Sp for metagenomics <0.90, classification based on ≥5 reads being positive was evaluated, followed by increments of five reads until a Sp of ≥0.90 was obtained for metagenomics.

As the prevalence of respiratory viruses was expected to differ between sample timing (arrival processing or approximately 2 weeks later) and age group (FPC or YRL), four populations were considered in the BLCM models (Figure 1): FPC sampled at arrival processing, YRL sampled at arrival processing, FPC sampled at 14 DOF, and YRL sampled at 14 DOF. Because the four population models failed to converge for BPIV-3, models using two populations based solely on the time of sampling were developed to estimate diagnostic test performance for the detection of BPIV-3 (Figure 1). For each model, covariance terms were included using default settings to account for potential correlation between the two nucleic acid-based tests (Figure 1). These models have (2*^R^* – 1)*S* degrees of freedom for *R* tests in *S* populations (63); accordingly, the four population models have 12 degrees of freedom while the two population models only have six degrees of freedom. Two population BPIV-3 models with and without covariance were, therefore, compared as a sensitivity analysis (Supplementary material 2).

Models were developed and run using JAGS software v4.3.0 (64) and the runjags package (65) in R v4.1.0 (66). Uninformative priors [beta(1,1)] were used for Se and Sp for metagenomic sequencing and qPCR, as well as the prevalence for each population. Convergence was deemed satisfactory for all models by evaluating model diagnostics, including potential scale reduction factor (<1.05), effective sample size (>1,000), and Monte Carlo standard errors as a percent of standard deviation (<5%), as well as visual inspection of trace, autocorrelation, empirical cumulative distribution function, and density plots (67, 68). Parameters were estimated using 50,000 iterations in each of three chains after a burn-in phase of 5,000 iterations per chain for most models. Models for BoHV-1 test performance were the exception; due to autocorrelation and a slightly low effective sample size at 50,000 iterations, models for this virus were run for 100,000 iterations in each of 3 chains after burn-in. Estimates for test diagnostic Se and Sp were reported as the median of the posterior along with 95% credible intervals (95% CrI). Samples were categorized as positive or negative based on the read count thresholds determined by the primary BLCM analysis (Figures 1, 2).

To explore the impact of different approaches for adjusting read counts to account for contamination/barcode crosstalk based on read counts for each virus in the water controls, the BLCM analyses were repeated without adjustment for the water controls and after adjusting for the median read counts for each virus in the water controls (Supplementary material 2). Estimates were considered significantly different from each other when the 95%CrI did not overlap.

To facilitate more direct comparison of results to existing estimates in the literature, and to evaluate potential differences associated with using Bayesian latent class models and biases in not using these models, diagnostic Se and Sp for metagenomic analyses were also calculated for the viruses using qPCR as a gold reference standard and the diagt program in Stata (StataCorp LLC, College Station, TX). Kappa summarizing agreement between metagenomic classification, qPCR and culture as appropriate was also calculated using Stata and reported in Supplementary material 2.

### 2.9 BLCM analyses for bacterial reads identified in the viral metagenomic pipeline

The bacterial reads from the viral nanopore metagenomic sequencing protocol used in this analysis are described in detail in the companion paper (21). BLCMs were created to evaluate read count-based detection cutoffs for four bacteria of interest concurrently identified in the viral metagenomic dataset: *Mannheimia haemolytica*, *Pasteurella multocida*, *Histophilus somni*, and *Mycoplasmopsis bovis* (formerly known as *Mycoplasma bovis*) (Supplementary Figure S3.1). The BLCMs used the same four populations (FPC arrival, YRL arrival, FPC 14 DOF, YRL 14 DOF) described for the virus data.

The BLCMs leveraged bacterial phenotype (culture) data that were also available from deep nasopharyngeal samples (DNPS) collected at the same times from the same animals (69). These culture data provided an opportunity to generate read count cutoff thresholds for detection of *M. haemolytica*, *P. multocida*, and *H. somni*, based on receiver operating characteristic (ROC) curves comparing the bacterial read counts to culture results. If Sp was <0.90, increments of one read were evaluated until a Sp of ≥0.90 was obtained for metagenomics (Supplementary Figure S3.1).

The DNPS collected from the companion study (21) were also tested using a qPCR assay for *M. bovis*, and a read count cutoff threshold for detection of *M. bovis* was generated based on a ROC comparing bacterial read counts to qPCR results (Supplementary Figure S3.1).

Laboratory methods specific to the bacterial analyses, including the culture and qPCR data used as the second test in the BLCM models and associated sensitivity analyses for the BLCM, are detailed in Supplementary material 3.

## 3 Results

### 3.1 Sample population

The nasal swab samples used in the BLCM analyses are described in the companion study (21). Briefly, 760 samples were collected: 520 from FPC across 13 pens and 240 from YRL across six pens, in 19 different feedlots. Twenty nasal samples were collected from the same pens at two distinct intervals: at arrival processing (time 1) and at a follow-up collection (time 2) between 10 and 23 DOF (median 14 DOF). The average arrival weight for the FPC pens was 266 kg with a standard deviation of 43 kg and for the YRL pens was 399 kg with a standard deviation of 66 kg.

### 3.2 Prevalence of six target viruses detected by qPCR

At least one of BCoV, BoHV-1, BPIV-3, BRSV, and IDV was identified using qPCR with the laboratory Ct threshold of ≤ 38.0 in 77.5% of the 760 samples from FPC and YRL. Based on qPCR, BCoV was the most prevalent (60.4%) virus detected in the 760 samples, followed by IDV (32.9%), BPIV-3 (19.5%), BoHV-1 (16.2%), and BRSV (13.6%) (Table 2). BVDV was not identified in any of the 760 samples using qPCR.

**Table 2:** Prevalence of six bovine respiratory disease viruses detected by quantitative reverse transcriptase polymerase chain reaction (qPCR) from 520 nasal swabs collected from 13 pens of fall placed calves (FPC) and 240 nasal swabs collected from 6 pens of yearlings (YRL) at arrival processing and at 14 days on feed (DOF) across 19 western Canadian feedlots.

Virus	Samples from FPC				Samples from YRL			
	Time 1: arrival (*n* = 260)		Time 2: 14 DOF (*n* = 260)		Time 1: arrival (*n* = 120)		Time 2: 14 DOF (*n* = 120)	
	Number positive	Prevalence	Number positive	Prevalence	Number positive	Prevalence	Number positive	Prevalence
BCoV	211	81.2%	152	58.5%	53	44.2%	43	35.8%
BoHV-1	46	17.7%	53	20.4%	7	5.8%	17	14.2%
BPIV-3	54	20.8%	82	31.5%	4	3.3%	8	6.7%
BRSV	6	2.3%	83	31.9%	3	2.5%	11	9.2%
BVDV	0	0%	0	0%	0	0%	0	0%
IDV	49	18.9%	143	55.0%	16	13.3%	42	35.0%

BCoV, bovine coronavirus; BoHV-1, bovine herpes virus; BPIV-3, bovine parainfluenza virus 3; BRSV, bovine respiratory syncytial virus; BVDV, bovine viral diarrhea virus 1 and 2; IDV, influenza D virus. Cut-off for positive results Ct ≤38.0 reported by commercial diagnostic laboratory (PDS, Saskatoon, SK).

The qPCR data were also summarized utilizing a Ct value of ≤ 37.0 as a cutoff for positive samples. Based on this alternative cutoff, the overall prevalence of each target virus was as follows: BCoV, 54.1%; IDV, 28.2%; BPIV-3, 14.3%; BoHV-1, 10.7%: BRSV, 10.4%; and BVDV 0%. The change in qPCR cutoff from ≤38.0 to ≤37.0 had the biggest relative difference for the interpretation of the BoHV-1 results. Thirty-two percent (39/123) of samples classified as positive for BoHV-1 by the regional diagnostic laboratory (PDS, Saskatoon, SK) had Ct values between 37 and 38.

### 3.3 BRD virus detection by metagenomic sequencing

As previously reported (21), of the total 841.6 million reads, the average sample quality score was 14.5 with an average length for all reads of 501 bp across samples. The median host DNA across samples was 77.7%. The 1.95 million viral reads represented 0.2% of total reads and the 3.4 million bacterial reads accounted for 0.4% of total reads.

To facilitate direct comparison to the qPCR results, the number of metagenomic sequencing reads for BVDV-1 and BVDV-2 were combined and reported as BVDV. Briefly, for the six individual viruses associated with BRD, the shortest total read length by sample varied from 215 bp for BVDV to 321 bp for BPIV-3, while the sum of Kraken sequence lengths across all samples ranged from 12,022 bp for BoHV-1 to 1,218,094,996 bp for BCoV (Table 3). The proportion of samples in which one or more reads of individual viruses were detected ranged from 52.0% for BCoV to 0.9% for BoHV-1 (Table 3).

**Table 3:** Sequence length characteristics for six viruses associated with bovine respiratory disease detected by metagenomic sequencing in 760 nasal samples collected from 13 pens of fall placed calves (FPC) and 6 pens of yearlings (YRL) in western Canadian feedlots.

Read length (nucleotides)	BCoV	BoHV-1	BPIV-3	BRSV	BVDV	IDV
Sum of all sequences (bp)	1,218,094,996	12,022	70,603,669	2,740,070	63,788	406,331,925
Median of classified total sequence per sample (bp)*	8,792	819	3,727	1730	1,006	8,683
Shortest classified sequence (bp)	219	242	321	224	215	225
Proportion of samples ≥1 sequence	52.0%	0.9%	33.7%	27.4%	5.1%	31.1%
Median sequences detected per sample *	10	1	4	3	1	9
Genome size (bp) for reference	30,845	134,896	15,537	15,140	12,573	12,546

BCoV, bovine coronavirus; BoHV-1, bovine herpes virus; BPIV-3, bovine parainfluenza virus 3; BRSV, bovine respiratory syncytial virus; BVDV, bovine viral diarrhea virus 1 and 2; IDV, influenza D virus. *Median reported for samples where the virus was detected in at least one read.

### 3.4 Sensitivity and specificity of BRD virus detection by qPCR and nanopore metagenomic sequencing based on Bayesian latent class models

A comparison of the sensitivities for both qPCR and nanopore metagenomic sequencing for the detection of each virus shows the diagnostic Se of qPCR was higher than nanopore metagenomic sequencing for the detection of BCoV and BoHV-1 (Table 4). The diagnostic Se of metagenomic sequencing, however, was higher than qPCR for detecting BRSV. No significant difference was noted in the Se of qPCR and metagenomics sequencing for the detection of BPIV-3 and IDV, as the 95% CrIs of each test overlapped. No diagnostic test performance estimates were available for the detection of BVDV as no qPCR positive samples were available for comparison in the BLCM.

**Table 4:** Sensitivity and specificity of quantitative reverse transcriptase polymerase chain reaction (qPCR) and metagenomic sequencing for the detection of five of six viruses associated with bovine respiratory disease in 760 nasal swabs collected from 13 pens of fall-placed calves (FPC) and 6 pens of yearlings (YRL) in western Canadian commercial feedlots based on the results of Bayesian latent class models (primary analyses).

Virus	Test	Sensitivity			Specificity		
		Lower95	Median	Upper95	Lower95	Median	Upper95
BCoV	qPCR	0.81	**0.90**	0.999	0.51	**0.59**	0.68
	Metagenomics ≥30 reads	0.25	**0.35**	0.46	0.88	**0.91**	0.95
BoHV-1	qPCR	0.19	**0.39**	0.99	0.85	**0.93**	0.996
	Metagenomics ≥1 read	0.01	**0.04**	0.15	0.99	**0.998**	0.999
BPIV-3	qPCR	0.21	**0.42**	0.66	0.80	**0.86**	0.92
	Metagenomics ≥5 reads	0.19	**0.52**	0.87	0.89	**0.94**	0.999
BRSV	qPCR	0.22	**0.32**	0.43	0.96	**0.98**	0.999
	Metagenomics ≥1 read	0.44	**0.60**	0.77	0.89	**0.94**	0.99
IDV	qPCR	0.53	**0.65**	0.79	0.84	**0.92**	0.999
	Metagenomics ≥1 read	0.48	**0.60**	0.73	0.83	**0.91**	0.98

BCoV, bovine coronavirus; BoHV-1, bovine herpes virus; BPIV-3, bovine parainfluenza virus 3; BRSV, bovine respiratory syncytial virus; IDV, influenza D virus; Lower95, lower band of 95% credible interval; Upper95, upper band of 95% credible interval; qPCR, quantitative reverse transcriptase polymerase chain reaction. Read numbers used in the primary analyses adjusted for means of water controls from each sequencing run (see Supplementary material 2 for sensitivity analysis for the unadjusted (S2.1a) and median adjusted (S2.1b) water controls). Estimates were not available for detection of BVDV (bovine viral diarrhea virus 1 and 2) as there were no qPCR positive samples. Samples were considered positive for a specific virus if the internal amplification control and both test duplicates gave a sigmoid amplification curve with a Ct (cycle threshold) value of ≤ 38.0. Median values are displayed in bold.

For the detection of BoHV-1, BRSV, and IDV, the Sp for metagenomic sequencing derived from BLCM analysis was ≥0.90 with a cutoff of ≥1 read for a positive classification (Figure 2; Table 4). However, the detection of BCoV and BPIV-3 required cutoffs set at ≥30 and ≥5 reads, respectively, to reach the Sp threshold of 0.90 (Figure 2). The Sp of qPCR and nanopore metagenomic sequencing was comparable for the detection of most viruses, except for BCoV, where nanopore metagenomics outperformed qPCR (Table 4).

This same pattern was observed for both Se and Sp estimates for the unadjusted and median water controls (Supplementary Table S2.1). Specifically, the 95% CrI overlapped between models without adjustment for the water controls (Supplementary Table S2.1a), models adjusted for the median for the water controls (Supplementary Table S2.1b), and models adjusted for the mean of the water controls (Table 4).

The impact of the covariance term was examined for BPIV-3 alone as it was the only model limited to two populations, resulting in fewer degrees of freedom to estimate covariance parameters. The BPIV-3 BLCM without covariance (Supplementary Table S2.2a) yielded comparable Se and Sp estimates to the model with covariance reported in Table 4. The 95% Crl for the estimates for the models with and without covariance overlapped, suggesting the model was not sensitive to the inclusion of the covariance term.

### 3.5 Prevalence of six viruses detected by nanopore metagenomic sequencing

At least one of BCoV, BoHV-1, BPIV-3, BRSV, BVDV, or IDV was identified by metagenomic sequencing in 60.3% of the 760 samples from FPC and YRL. Using the read count positive cutoffs informed by BLCM models, IDV (31.1%) was the most prevalent virus detected across all 760 samples, followed by BRSV (27.4%) and BCoV (18.9%) (Table 5). Bovine parainfluenza virus 3 was identified in 14.7% of all samples and BoHV-1 in 0.9%. Using the default value of ≥1 read, BDVD-1 or BVDV-2 were identified in 5.1% of all samples.

**Table 5:** Prevalence of six respiratory viruses detected by metagenomics sequencing in 520 nasal swabs collected from 13 pens of fall-placed calves (FPC) and 240 nasal swabs collected from 6 pens of yearlings (YRL) at arrival processing and at 14 days on feed (DOF) across 19 western Canadian feedlots.

Virus	Samples from FPC				Samples from YRL			
	Time 1: arrival		Time 2: 14 DOF		Time 1: arrival		Time 2: 14 DOF	
	(*n* = 260)		(*n* = 260)		(*n* = 120)		(*n* = 120)	
	Number positive	Prevalence	Number positive	Prevalence	Number positive	Prevalence	Number positive	Prevalence
Cutoffs informed by BLCM								
BCoV reads ≥30	82	32%	27	10%	14	12%	21	18%
IDV reads ≥1	44	17%	133	51%	19	16%	40	33%
BRSV reads ≥1	22	8.5%	67	26%	47	39%	72	60%
BPIV-3 reads ≥5	11	4.2%	30	12%	24	20%	47	39%
BoHV-1 reads ≥1	0	0.0%	7	2.7%	0	0.0%	0	0.0%
Cutoff not informed by BLCM								
BVDV reads ≥1	3	1.2%	4	1.5%	10	8.3%	22	18%

BCoV, bovine coronavirus; BoHV-1, bovine herpes virus; BRSV, bovine respiratory syncytial virus; BPIV-3, bovine parainfluenza virus 3; BVDV, bovine viral diarrhea virus 1 and 2; IDV, influenza D virus; BLCM, Bayesian latent class model. Read numbers used in analyses were adjusted for means of water controls from each sequencing run.

### 3.6 Impact of changing the read count threshold on diagnostic test performance of metagenomic sequencing and qPCR and relative prevalence by metagenomic sequencing

The impact of an alternative cutoff of ≥1 read on diagnostic test performance and associated prevalence estimates was determined for BCoV and BPIV-3 (Tables 6, 7). For detection of BCoV, the Se of metagenomic sequencing was significantly higher and the Sp significantly lower with a cutoff of ≥1 read (Table 6) was than with an alternative cutoff of ≥30 reads (Table 4) No significant difference was noted in the Se or Sp of qPCR when metagenomic cut-off thresholds differed for BCoV (Tables 4, 6).

**Table 6:** Sensitivity and specificity of quantitative reverse transcriptase polymerase chain reaction (qPCR) and metagenomic sequencing based on BLCM cutoffs of at least 1, 5, or 30 reads per sample in 760 nasal swabs collected from 13 pens of fall placed calves (FPC) and 6 pens of yearlings (YRL) in western Canadian commercial feedlots.

Virus	Test	Sensitivity			Specificity		
		Lower95	Median	Upper95	Lower95	Median	Upper95
BCoV	qPCR	0.81	0.92	0.999	0.63	0.80	0.999
	Metagenomics ≥1 read	0.52	0.59	0.67	0.49	0.57	0.66
	qPCR	0.80	0.88	0.999	0.59	0.69	0.79
	Metagenomics ≥5 reads	0.37	0.44	0.55	0.74	0.80	0.86
	**qPCR**	**0.81**	**0.90**	**0.999**	**0.51**	**0.59**	**0.68**
	**Metagenomics ≥30 reads**	**0.25**	**0.35**	**0.46**	**0.88**	**0.91**	**0.95**
BoHV-1	**qPCR**	**0.19**	**0.39**	**0.99**	**0.85**	**0.93**	**0.996**
	**Metagenomics ≥1 read**	**0.01**	**0.04**	**0.15**	**0.99**	**0.998**	**0.999**
	qPCR	0.20	0.41	0.999	0.88	0.94	0.999
	Metagenomics ≥5 reads	0.00	0.02	0.08	0.99	0.998	0.999
	qPCR	No samples with ≥30 BoHV-1 reads					
	Metagenomics ≥30 reads						
BPIV-3	qPCR	0.21	0.46	0.86	0.81	0.87	0.96
	Metagenomics ≥1 read	0.36	0.63	0.94	0.66	0.73	0.84
	**qPCR**	**0.21**	**0.42**	**0.66**	**0.80**	**0.86**	**0.92**
	**Metagenomics ≥5 reads**	**0.19**	**0.52**	**0.87**	**0.89**	**0.94**	**0.999**
	qPCR	0.30	0.73	0.999	0.81	0.87	0.96
	Metagenomics ≥30 reads	0.04	0.22	0.50	0.97	0.99	0.999
BRSV	**qPCR**	**0.22**	**0.32**	**0.43**	**0.96**	**0.98**	**0.999**
	**Metagenomics ≥1 read**	**0.44**	**0.60**	**0.77**	**0.89**	**0.94**	**0.99**
	qPCR	0.23	0.40	0.67	0.97	0.99	0.999
	Metagenomics ≥5 reads	0.14	0.25	0.42	0.97	0.99	0.999
	qPCR	0.33	0.69	0.999	0.97	0.99	0.999
	Metagenomics ≥30 reads	0.02	0.06	0.13	0.99	0.996	0.999
IDV	**qPCR**	**0.53**	**0.65**	**0.79**	**0.84**	**0.92**	**0.999**
	**Metagenomics ≥1 read**	**0.48**	**0.60**	**0.73**	**0.83**	**0.91**	**0.98**
	qPCR	0.54	0.70	0.88	0.82	0.87	0.94
	Metagenomics ≥5 reads	0.27	0.39	0.52	0.95	0.98	0.999
	qPCR	0.55	0.77	0.96	0.81	0.86	0.91
	Metagenomics ≥30 reads	0.15	0.25	0.36	0.98	0.99	0.999

BCoV, bovine coronavirus; BoHV-1, bovine herpes virus; BRSV, bovine respiratory syncytial virus; BPIV-3, bovine parainfluenza virus 3; BVDV, bovine viral diarrhea virus 1 and 2; IDV, influenza D virus; BLCM, Bayesian latent class model; Upper95, upper band of 95% credible interval; qPCR, quantitative reverse transcriptase polymerase chain reaction. Read numbers used in the analysis were adjusted for mean of water controls from each sequencing run. Samples were considered positive for a specific virus if the internal amplification control and both test duplicates gave a sigmoid amplification curve with a Ct (cycle threshold) value of ≤ 38.0. Final models used in the analysis are bolded.

**Table 7:** Prevalence of two viruses associated with bovine respiratory disease detected by metagenomics sequencing based on an alternative BLCM cutoff of at least 1 read per sample from 520 nasal swabs collected from 13 pens of fall-placed calves (FPC) and 240 nasal swabs collected from 6 pens of yearlings (YRL) at arrival processing and at 14 days on feed (DOF) across 19 western Canadian feedlots.

Virus	FPC				YRL			
	Time 1: arrival		Time 2: 14 DOF		Time 1: arrival		Time 2: 14 DOF	
	(*n* = 260)		(*n* = 260)		(*n* = 120)		(*n* = 120)	
	Number positive	Prevalence	Number positive	Prevalence	Number positive	Prevalence	Number positive	Prevalence
BCoV read ≥1	154	59%	127	49%	52	43%	62	52%
BPIV-3 read ≥1	42	16%	73	28%	68	57%	73	61%

BCoV, bovine coronavirus; BPIV-3, bovine parainfluenza virus 3; DOF, days on feed. Read numbers used in analyses were adjusted for means of water controls from each sequencing run.

For the detection of BPIV-3, no significant difference was noted in the Se of metagenomic sequencing between alternative cutoffs of ≥1 read or ≥5 reads (Tables 4, 6). However, the Sp of metagenomic sequencing was significantly lower with a cutoff of ≥1 read (Table 6) than with an alternative cutoff of ≥ 5 reads (Table 4). No significant difference was noted in the performance of qPCR for the detection of BPIV-1 with metagenomic cutoffs of ≥1 read or ≥5 reads (Tables 4, 6). For metagenomic data classified based on ≥1 read (Table 7), the prevalences of BCoV and BPIV-3 were substantially higher than when based on BLCM cutoffs (Table 5). Regardless of the read count cutoff, most trends in the prevalences of BCoV and BPIV-3 between samples from FPC and YRL and samples collected at arrival processing and 14 DOF were similar (Tables 5, 7).

Table 6 also contains a complete Se analysis of the impact of varying the cutoff thresholds for BoHV-1, BRSV, and IDV.

### 3.7 Impact of estimating test performance for metagenomics assuming qPCR as a gold standard

In an alternative calculation, with a more traditional approach assuming qPCR as the gold reference standard, the estimated Se of metagenomics was at least 10% lower for evaluating BCoV, BPIV-3, and BRSV (Table 8) than the median values generated by the BLCMs (Table 4); however, the differences were not statistically significant. Furthermore, the estimated Sp of the metagenomics for BoHV-1 and BRSV were significantly lower based on the assumption of qPCR as a gold standard compared to BLCMs (Table 8). All other differences were minimal.

**Table 8:** Sensitivity and specificity of metagenomic sequencing for the detection of six viruses associated with bovine respiratory disease in 760 nasal swabs collected from 13 pens of fall-placed calves (FPC) and 6 pens of yearlings (YRL) in western Canadian commercial feedlots.

Virus	Test	Sensitivity			Specificity		
		Lower95	Median	Upper95	Lower95	Median	Upper95
BCoV	Metagenomics ≥30 reads	0.20	**0.24**	0.28	0.85	**0.89**	0.92
BoHV-1	Metagenomics ≥1 read	0.02	**0.05**	0.10	0.50	**0.52**	0.54
BPIV-3	Metagenomics ≥5 reads	0.17	**0.23**	0.31	0.84	**0.87**	0.90
BRSV	Metagenomics ≥1 read	0.32	**0.42**	0.52	0.71	**0.75**	0.78
BVDV*	Metagenomics ≥1 read	Estimates not available			0.93	**0.95**	0.96
IDV	Metagenomics ≥1 read	0.50	**0.57**	0.63	0.78	**0.82**	0.85

*Sensitivity estimates were not available for detection of BVDV (bovine viral diarrhea virus 1 and 2) as there were no qPCR positive samples. As qPCR was not targeted to BVDV-1 or BVDV-2 the reported estimate reflects the combination of both. BCoV, bovine coronavirus; BoHV-1, bovine herpes virus; BPIV-3, bovine parainfluenza virus 3; BRSV, bovine respiratory syncytial virus; IDV, influenza D virus; Lower95, lower 95% confidence interval; Upper95, upper 95% confidence interval; qPCR, quantitative reverse transcriptase polymerase chain reaction. Read numbers used in analyses were adjusted for means of water controls from each sequencing run. All calculations assumed quantitative reverse transcriptase polymerase chain reaction (qPCR) was the gold reference standard (secondary analysis). Median values are displayed in bold.

### 3.8 Estimated positive and negative predictive value derived from diagnostic sensitivity and specificity estimates informed by the Bayesian latent class models

**Figure 3:**
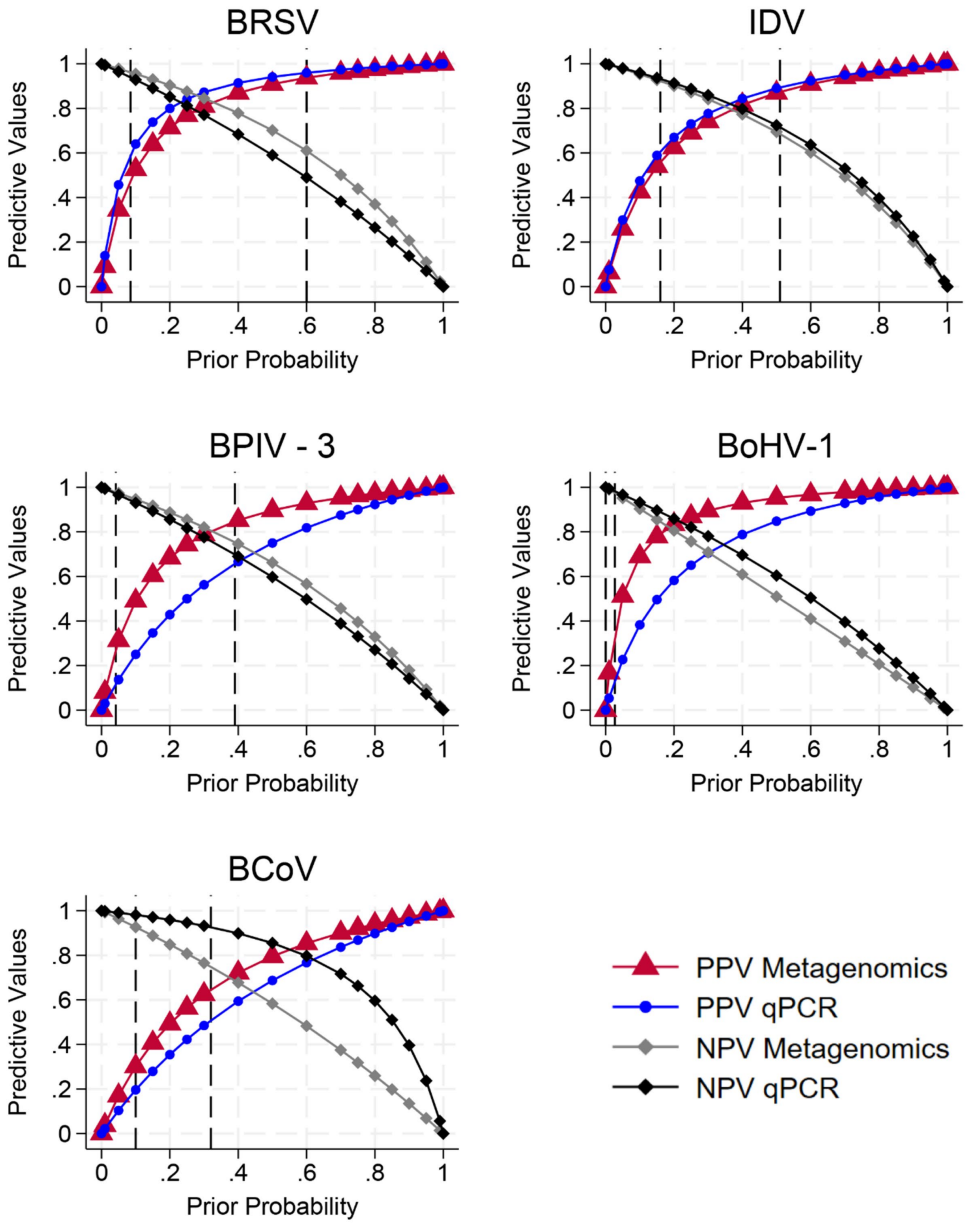
Positive and negative predictive values estimated for a range of pretest probabilities of disease based on the estimates of diagnostic sensitivity and specificity informed by Bayesian latent class models for both viral metagenomics and quantitative polymerase chain reaction. The vertical lines in each figure represent the upper and lower prevalence of each virus described for fall-placed calves and yearlings at arrival and 14 days on feed (Table 5) to highlight the areas of pretest probability most likely to be relevant for clinical interpretation. BCoV, bovine coronavirus; BoHV-1, bovine herpes virus; BRSV, bovine respiratory syncytial virus; BPIV-3, bovine parainfluenza virus 3; IDV, influenza D virus; BLCM, Bayesian latent class model; qPCR, quantitative polymerase chain reaction. BLCM estimates of diagnostic test sensitivity and specificity were not available for BVDV, bovine viral diarrhea virus types 1 and 2 as there were no qPCR positive samples.

Positive and negative predictive values were very similar between metagenomics and qPCR across a wide range of pretest probabilities of disease (Figure 3). In the range of pretest probabilities corresponding to the highest and lowest IDV prevalence reported in the present study (Table 5), estimated PPV and NPV were informative for both assays. The results for other assays varied more (Figure 3) with PPV being particularly limited within the range of expected prevalences for BoHV-1 and BCoV, but assay diagnostic Se being so low for some assays that the assay contributed very little beyond the very low pre-test probability to the NPV.

### 3.9 Sensitivity and specificity of BRD bacteria detection by viral nanopore sequencing using BLCMs

Characteristics for select respiratory bacterial species concurrently identified in the viral metagenomic dataset for which either bacterial culture or qPCR comparative data were also available are summarized in Table 9.

**Table 9:** Read characteristics of select respiratory bacteria concurrently identified in the viral metagenomic dataset derived from 760 nasal samples collected from 13 pens of fall placed calves (FPC) and 6 pens of yearlings (YRL) in western Canadian feedlots.

Read length (nucleotides)	*Mannheimia haemolytica*	*Pasteurella multocida*	*Histophilus somni*	*Mycoplasmopsis bovis*
Sum of all Kraken classified sequence lengths (bp)	5.18E+07	6.81E+06	1.22E+06	4.44E+06
Median of Kraken classified total sequence lengths per sample (bp)*	3,166	1846	479	2,444
Shortest Kraken classified sequence length per sample (bp)	207	209	200	200
Proportion of samples ≥1 sequence detected	59%	41%	67%	5%
Median sequences (reads) detected per sample *	38	8	6	4
Genome size (Mbp) for reference	2.8	2.3	2.3	1.0

*Median reported for samples where the virus was detected in at least one read.

Detection of all bacteria except *M. bovis* required establishing an ROC-/BLCM-based cutoff greater than 1 read to reach a minimum Sp of 0.90 (Table 10; Supplementary Table S3.1). The ROC-based cutoff of ≥8 reads optimizing both Se and Sp for the detection of *H. somni* resulted in a Sp above 0.90 (Table 10; Supplementary Table S3.1). However, the initial ROC-based cutoffs of ≥9 reads for the detection of *M. haemolytica* and ≥5 reads for the detection of *P. multocida* did not result in a Sp above 0.90 (Supplementary Tables S3.1, S3.2). In the final models, the read count cutoff was increased from ≥9 to ≥14 reads for the detection of *M. haemolytica* and from ≥5 to ≥9 reads for the detection of *P. multocida* (Table 10).

**Table 10:** Sensitivity and specificity of viral metagenomic sequencing compared to bacterial culture or quantitative reverse transcriptase polymerase chain reaction (qPCR) for the detection of four bacteria associated with bovine respiratory disease in 760 nasal swabs collected from 13 pens of fall placed calves (FPC) and 6 pens of yearlings (YRL) in western Canadian commercial feedlots.

Bacteria	Test	Sensitivity			Specificity		
		Lower95	Median	Upper95	Lower95	Median	Upper95
*M. haemolytica*	Metagenomics ≥14 reads	0.51	**0.64**	0.84	0.77	**0.90**	0.999
	Culture	0.27	**0.34**	0.42	0.83	**0.91**	0.999
*P. multocida*	Metagenomics ≥9 reads	0.35	**0.50**	0.68	0.86	**0.90**	0.95
	Culture	0.58	**0.79**	0.999	0.86	**0.92**	0.99
*H. somni*	Metagenomics ≥8 reads	0.54	**0.71**	0.87	0.86	**0.92**	0.99
	Culture	0.11	**0.17**	0.25	0.98	**0.999**	0.999
*M. bovis*	Metagenomics ≥1 read	0.31	**0.45**	0.61	0.96	**0.98**	0.999
	qPCR	0.44	**0.61**	0.81	0.92	**0.96**	0.99

Lower95, lower band of 95% credible interval; Upper95, upper band of 95% credible interval; qPCR, quantitative reverse transcriptase polymerase chain reaction (see Supplementary material 3 for more details). Read numbers used in the analyses adjusted for mean read numbers for each target from water controls from each sequencing run. Median values are displayed in bold.

When the sensitivities for detection of respiratory bacteria by both the viral metagenomic sequencing protocol and culture or qPCR were compared, the diagnostic Se of the viral metagenomic sequencing protocol was higher than culture for the detection of *M. haemolytica* and *H. somni* (Table 10). The CrIs overlapped for the sensitivities for the detection of both *P. multocida* and *M. bovis*. The specificities of viral nanopore metagenomic sequencing, culture, and qPCR were similar for detecting all selected respiratory bacteria (Table 10).

## 4 Discussion

Five viruses (BCoV, BoHV-1, BPIV-3, BRSV, and IDV) commonly associated with BRD were identified using both a qPCR assay and nanopore metagenomic sequencing of nasal swab samples collected from FPC and YRL in commercial feedlots in western Canada during arrival processing and again 2 weeks later. The results of these two diagnostic tests provided the data necessary to estimate test performance for samples collected under field conditions in which samples were collected in remote settings and then subjected to typical shipping delays to reach the laboratory. None of the nasal samples were qPCR positive for BVDV, limiting inferences on test performance. To the best of our knowledge, this is the first study to report the use of BLCMs to estimate the diagnostic performance (Se and Sp) of metagenomic sequencing and qPCR, in the absence of a gold standard, for detection of viruses associated with BRD in nasal samples.

Although the overall sensitivities of both nanopore metagenomic sequencing and qPCR were moderate to low for the detection of most viruses of interest, qPCR was more sensitive for the detection of BoHV-1 and BCoV than metagenomic sequencing. Metagenomic sequencing was more sensitive for identifying BRSV than qPCR. No significant difference in test sensitivities was noted for detection of BPIV-3 and IDV. Additionally, the two laboratory methods demonstrated comparable Sp in the detection of four of the five viruses with BLCM-informed cutoffs, while nanopore metagenomic sequencing exhibited higher Sp than qPCR for the detection of BCoV from nasal samples. Compared to qPCR, which targets short sequences of 50–300 base pairs, nanopore sequencing can generate reads up to several megabases in length, enabling the resolution of complex genomic regions and full-length transcripts, thereby enhancing diagnostic Sp (70). In these samples (21), median read lengths for targets of interest varied from 412 to 934 bp, with median read lengths for 18 of 21 virus >600 bp and individual read lengths for some viruses exceeding 2,700 bp. While the two tests varied in their ability to detect individual viruses, nanopore metagenomic sequencing offered a potential alternative for diagnostic laboratories to identify three of the five most important BRD viruses in this study population.

The sole other study comparing qPCR and two metagenomics sequencing platforms (MiSeq and nanopore) for the detection of IDV in respiratory tract samples assumed qPCR as the gold standard (36). This previous study used the IDV qPCR adapted by the diagnostic lab for this study (36). While complete in-house validation was available for adaptations to the other qPCR assays, information on the qPCR for IDV was more limited (36) and additional validation work is recommended. Zhang et al. reported agreement between the IDV qPCR and nanopore metagenomic sequencing of 57.9%, and Se and Sp for MiSeq metagenomic sequence detection relative to qPCR of 28.3 and 98.9%, respectively (36). In the present study, qPCR and nanopore metagenomics both demonstrated comparable Se and Sp in the identification of IDV, exceeding the Se value for MiSeq analysis reported in the earlier study.

While no previous studies have utilized BLCMs to assess the performance of diagnostic tests for the detection of multiple viruses associated with BRD in Canadian feedlot cattle, others have applied BLCM to examine test performance for other infections in Canadian cattle (33, 71). Additionally, researchers in Belgium employed BLCMs to assess various methods for detecting *M. bovis*, a bacterial pathogen associated with BRD in bronchioalveolar lavage samples; these methods included nanopore metagenomic sequencing, triplex qPCR assay, and matrix-assisted laser desorption ionization-time of flight (MALDI-TOF) mass spectrometry (37). The Se (77.3, 95% CrI 57.8–92.8%) and Sp (97.4, 95%CrI 91.5–99.7%) Bokma et al. (37) estimated using BLCMs for nanopore metagenomic sequencing overlapped with those reported for *M. bovis* in the present study. This was particularly true for the performance estimates reported for the raw reads in Supplementary material 3 (Se 69% (95%CrI 52–85%); Sp 98% (95%CrI 96–99.9%)), where the analysis was most similar to Bokma et al. (37).

One of the most notable aspects of the present analysis was the estimation of test performance for the detection of both viruses and bacteria utilizing a metagenomic protocol optimized for the detection and characterization of viruses. In addition to estimating Se and Sp for the detection of *M. bovis*, the present study estimated diagnostic Se for the detection of *M. haemolytica*, *P. multocida*, and *H. somni*. The number of reads and total base pairs of each bacterial species of interest detected in this study were limited compared to previous reports that utilized metagenomic protocols optimized for the detection and characterization of bacteria (58, 72). However, the estimated sensitivities of the viral nanopore metagenomic sequencing protocol in the present study were equivalent to culture for the detection of *P. multocida* and better than culture for the detection of *M. haemolytica* and *H. somni*. No significant differences were noted in the specificities between these two testing modalities for the same bacteria.

While virus isolation and bacterial culture, despite their inherent limitations, remain the gold standard for BRD pathogen detection, molecular assays such as conventional and real-time PCR are more commonly used in veterinary diagnostic laboratories, particularly for virus detection, due to speed, efficiency, Se, and Sp (73, 74). The imperfect Se and Sp estimates for qPCR generated by this study highlight that using PCR as a gold standard could bias Se and Sp estimates for other tests and supports the use of BLCMs to estimate diagnostic test performance (28, 75). In the secondary analysis reported here in which qPCR was used as gold standard, Sp was significantly underestimated for BoHV-1 and BRSV. Sensitivity was also underestimated, but the difference was not statistically significant, for BCoV, BPIV-3, and BRSV.

Several factors can impact the Se and Sp of qPCR. A meta-analysis by Barnewall et al. (76) on qPCR for BRD diagnosis revealed the effectiveness of qPCR detection is dependent on both the Sp of primer-probe sequences used for identification and the choice of target gene. For the present study, BLAST results for the primers used in the qPCR, and reported in Supplementary material 1, suggested a generally high level of Sp. Moreover, the continuous evolution of pathogens and updates to pathogen sequence databases can influence qPCR assay detection Se because every qPCR assay was limited by the data that was in hand when the primers and probes were designed (77). For example, the primer used for the commercial qPCR for BCoV targets the M gene (43). However, despite the high proportion of previously reported whole genomes identified by our in-silico analysis, it is likely that this gene does not capture all possible circulating regional strains given the limited agreement between metagenomics and results of the BLAST analysis. Furthermore, in cases where multiple similar pathogens are present, such as in respiratory samples, qPCR assay Se can be affected due to potential cross-reactivity (78). The accuracy of qPCR results can be further impacted by the quality and quantity of sample DNA or RNA, highlighting the importance of proper sample handling and preparation (79). Additionally, qPCR is prone to false negative results, when quantifying less abundant targets or when sample inhibitors interfere with the reaction, and to false positive results should the sample or analytical process be contaminated (80).

The Se and Sp of metagenomic sequencing for viral detection and characterization can also be influenced by several factors. The samples in the present study were collected as part of routine surveillance activities in low to high-risk animals; however, no effort was made to target animals sick with BRD. As such, the number of virus particles expected in many of these samples would be expected to be low, or at least lower than in animals at or immediately prior to clinical disease. In clinical specimens with high levels of host DNA, metagenomics can encounter difficulties detecting pathogens in small quantities within low microbial biomass or in complex sample matrices (81). In the present study, even after host depletion, a median of 78% of host DNA across samples was noted in the final data. The Se of these assays could improve substantially if the protocol for removal of host DNA before sequencing could be further optimized. The depth of sequencing, or total number of base pairs sequenced per sample, also directly impacts metagenomic Se, with deeper sequencing generally enabling the detection of less abundant viruses, albeit at a higher expense (82, 83).

Limited standardization of bioinformatics protocols, analysis tools, and reference databases can also affect the Se and Sp of metagenomic testing and present computational challenges. Additionally, the vast amount of data generated by metagenomics can sometimes complicate interpretation (82), as can the definition of the read count threshold used to classify a sample as positive. In this study we set the read count threshold based on achieving a minimum Sp of ≥0.90, which resulted in a higher read count threshold for BCoV and BPIV-3 compared to the other viruses. Defining the threshold to classify a test as positive always necessitates balancing Se and Sp for test utility, and prioritizing Sp introduces the potential to sacrifice Se. This was particularly evident in the case of BCoV, where the read threshold required to reach our minimum Sp (≥30 reads) significantly reduced Se when compared to estimates using a threshold of one read. Cross-barcode contamination can also impact Sp, potentially leading to false-positive results. Increased multiplexing could further diminish the Se and accuracy of the assay (36). In the present study, library preparation water controls were added to every flow cell and the results used to adjust the sample results and reduce any impact of cross-barcode contamination. Further, all samples from one pen were analyzed together on a single flow cell. As such, any residual impacts of cross-barcode contamination would be limited to samples within the same pen.

BLCMs incorporate true disease status as a latent variable and typically use dichotomous outcomes from multiple diagnostic tests to estimate the respective sensitivities and specificities (26). Incorporating results from multiple populations with different prevalences, in addition to multiple tests, increases degrees of freedom and model identifiability to estimate parameters of interest, including the Se and Sp of each test. The three key assumptions are: (1) varied disease prevalence among subpopulations, (2) constant Se and Sp within subpopulations, and (3) conditional independence of tests (26). In this study, our four study subpopulations were FPC and YRL at arrival processing and at 2 weeks on feed; differences in the prevalence of viruses in these subpopulations are documented in the companion paper (21).

The independence of tests—a key assumption of BLCMs—could not be presumed in the current analysis as both tests utilized information which is based upon the population of DNAs and RNAs in the sample. Consequently, covariance terms were incorporated into the BLCMs. The advantage of BLCMs over other latent class approaches for estimating test performance, in the absence of a gold standard, lies in their ability to adjust for and estimate the extent of covariance among diagnostic test results by directly including terms in the model, provided sufficient degrees of freedom (28). Because the models for BPIV-3 did not converge when four populations were used, models with two populations were developed, which limited degrees of freedom to include covariance terms. To investigate the impact of including covariance terms in this case, models with and without covariance terms were compared and shown to result in similar test performance estimates. While median estimates varied, credible intervals considerably overlapped for Se in both model configurations. In addition, a range of model outputs related to model fit were reported for all models from this study. An evaluation of potential scale reduction factor, effective sample size, Monte Carlo standard errors as a percentage of standard deviation, and visual inspection of trace, autocorrelation, empirical cumulative distribution function, and density plots ensured criteria for convergence were met.

As noted previously, one potential limitation of this study is the relatively low prevalence of some target viruses, resulting in smaller differences among the four study populations used to inform the BLCMs for some viruses. Given the study was based on a two-test model where the tests were potentially correlated, this additional challenge could have further restricted the information available to generate test performance estimates. While the model diagnostics were good, the few test-positive results might have impacted the precision, and potentially the point estimates of Se, and contributed to wide 95% CrIs as seen for BoHV-1.

The effect of low prevalence on estimating diagnostic test Se can be complex according to Murad et al. (84). They conducted a study using mixed-effects random-intercept linear regression models to examine the relationship between prevalence and logit-transformed Se and Sp. Their research, based on data from meta-analyses of diagnostic test accuracy and published in the Cochrane Database of Systematic Reviews (2003–2020), revealed that higher prevalence was associated with increased estimated Se (84). The study found that, compared to the lowest prevalence quartile, the upper quartiles showed significantly higher odds of identifying true positive cases, with odds ratios ranging from 1.17 to 1.47 (84). These findings indicate that, in settings with low prevalence, the estimated Se of a diagnostic test can be lower.

The limited sensitivities of these assays have the greatest potential to impact the NPV or the clinician’s confidence in being able to rule out the presence of the virus. In the present study, test diagnostic Se and Sp were considered together with observed prevalence of these viruses to estimate both PPV and NPV for each assay. For example, despite the relatively low Se for both the IDV and BRSV assays, the NPV was >70% with the exception of FPC at 14 DOF for IDV and YRL at 14 DOF for BRSV. However, some of the tests, such as BoHV-1, had diagnostic Se that were so low that the tests contributed little certainty beyond the pre-test probability to the estimated NPV. In addition, at the lower range of prevalences observed for some viruses such as BoHV-1, PPV was also limited even those tests where diagnostic Sp was considerably higher than Se.

As technologies continue to evolve and improve base pair quality, the Se of viral nanopore metagenomic sequencing is expected to continue to improve. Nanopore sequencing libraries have some propensity to create duplex reads which could contribute to an improvement in accuracy for individual reads. Improved base pair quality could then potentially improve Se by reducing the proportion of unclassified reads. Following the completion of this sequencing, ONT rolled out the Dorado basecaller as an alternative to Guppy. This newer basecaller leverages neural network architecture and has shown improved accuracy with the flow cells and library kits reported in the present study (85).

Despite the current limitations of these technologies, nanopore metagenomic sequencing has the potential to contribute important information not just for the viruses targeted in the present study but others, such as bovine rhinitis B virus, not routinely assayed by commercial qPCR tests (21). Nanopore metagenomic sequencing also provides the potential to complete viral and even bacterial assemblies to better describe identified pathogens when sufficient sequencing depth is achieved (86). Further, other nanopore metagenomic sequencing protocols provide the potential for identifying antimicrobial resistance genes within raw reads of bacterial species of interest to BRD where coverage and read length are sufficient (87, 88), unlike the present methodology that simply identified antimicrobial resistance genes but did not link them to BRD bacteria of interest.

The integration of metagenomics into everyday clinical use faces several obstacles, including the requirement for reliable data analysis protocols as no universally accepted bioinformatics pipeline can currently perform a fast, sensitive, and precise analysis (79, 89, 90). As technology progresses and costs per sample decrease, metagenomics are expected to play an increasingly significant role in infectious disease management. However, the challenges associated with implementing clinical metagenomics extend beyond technical aspects (91) to include practical (92) and regulatory considerations (93). As research continues to advance, efforts are being made to address these hurdles and establish standardized protocols for metagenomic diagnostics (94, 95) as well as methods for evaluating test performance. Overcoming these obstacles could potentially revolutionize clinical microbiology, offering comprehensive and rapid diagnostic solutions for complex infections, syndromes, and microbiome-related disorders.

## 5 Conclusion

While nanopore metagenomic sequencing and quantitative polymerase chain reaction (qPCR) methods both exhibited generally low to moderate Se in detecting BRD-associated viruses, they also both demonstrated high Sp. The detection of respiratory bacteria by the metagenomic protocol optimized for the detection and characterization of viruses was either comparable to qPCR and culture or exceeded culture. The appeal of metagenomic sequencing lies in comprehensive pathogen detection and characterization compared to qPCR. Further advancements in metagenomic sequencing in areas such as single-nucleotide variant detection and accuracy of base calling could lead to improved Se and overall effectiveness in viral detection.

## Data Availability

The genomic data used in this study have been deposited in the Sequence Reach Archive (SRA) within BioProject ID: PRJNA1374179. Custom scripts can be accessed at: https://github.com/coadunate/ASSETS_2 (Accessed October 26, 2025).

## Supplementary material

SUPPLEMENTARY MATERIAL 1 Summary of quality assurance information for qPCR completed by Prairie Diagnostic Services Inc., Saskatoon, SK Canada.

SUPPLEMENTARY MATERIAL 2 Assessment of BRD virus detection by nanopore sequencing and qPCR using Bayesian latent class models (BLCMs) - sensitivity analysis and kappa statistics.

SUPPLEMENTARY MATERIAL 3 Assessment of BRD bacteria detection by nanopore sequencing (protocol optimized for virus detection), culture and qPCR using Bayesian latent class analysis: Methods and supplemental results.
