# Supplementary material for: Diagnostic sensitivity and specificity of metagenomic sequencing and qPCR for detection of viruses associated with bovine respiratory disease estimated using Bayesian latent class models
Source: Front Vet Sci. 2026 Feb 25;13:1704414. doi: 10.3389/fvets.2026.1704414 (PMC12976743; doi:10.3389/fvets.2026.1704414)
Supplement: SUPPLEMENTARY MATERIAL 2 — Assessment of BRD virus detection by nanopore sequencing and qPCR using Bayesian latent class models (BLCMs) - sensitivity analysis and kappa statistics. [file Supplementary_file_2.pdf]

Diagnostic sensitivity and specificity of metagenomic sequencing and qPCR for detection of viruses associated with bovine respiratory disease estimated using Bayesian latent class models

Supplementary Material 2:

Assessment of BRD virus detection by nanopore sequencing and qPCR using Bayesian latent class models (BLCMs) - sensitivity analysis and kappa statistics

Outline:

Supplement A: Sensitivity analysis examining the impact of different options for adjusting for the results of the water controls on the analysis (Part 1 and 2)

Supplement B: Sensitivity analysis examining the impact of covariance terms in the BLCM model for detection of BPIV-3

Supplement C: Kappa calculations nanopore sequencing, qPCR and culture – respiratory viruses and bacteria

Supplement A: Part 1 of the sensitivity analysis examining the impact of different options for adjusting for the results of the water controls on the analysis

Table S1a. Bayesian latent class models using viral data that were not adjusted for virus data from the water controls.

Models compare qPCR and nanopore metagenomics to estimate the sensitivity and specificity of each diagnostic test for detection of viruses associated with BRD in 760 nasal samples collected from commercial feedlot cattle.

Virus	Test	Sensitivity			Specificity		
		Lower95	Median	Upper95	Lower95	Median	Upper95
**BCoV**	PCR	0.81	**0.92**	0.999	0.50	**0.56**	0.63
	Metagenomics reads ≥ 30	0.37	**0.49**	0.61	0.87	**0.91**	0.94
**BoHV-1**	PCR	0.19	**0.41**	0.99	0.85	**0.93**	0.995
	Metagenomics reads ≥ 1	0.01	**0.04**	0.16	0.99	**0.998**	0.999
**BPIV-3**	PCR	0.21	**0.40**	0.60	0.81	**0.86**	0.94
	Metagenomics reads ≥ 5	0.24	**0.59**	0.91	0.87	**0.93**	0.999
**BRSV**	PCR	0.21	**0.31**	0.41	0.96	**0.99**	0.999
	Metagenomics reads ≥ 1	0.47	**0.64**	0.81	0.89	**0.94**	0.99
**IDV**	PCR	0.52	**0.62**	0.71	0.82	**0.89**	0.94
	Metagenomics reads ≥ 1	0.71	**0.83**	0.93	0.80	**0.89**	0.97

Abbreviations:

BCoV: bovine coronavirus; BoHV-1: bovine herpes virus; BPIV-3: bovine parainfluenzavirus 3; BRSV: bovine respiratory syncytial virus; BVDV: bovine viral diarrhea virus 1 and 2; IDV: influenza D virus; Lower95: lower band of 95% credible interval; Upper95: upper band of 95% credible interval; qPCR: quantitative polymerase chain reaction.

Estimates were not available for detection of BVDV (bovine viral diarrhea virus 1 and 2) as there were no qPCR positive test results.

Sensitivity: PCR>Metagenomics = BCoV, BoHV-1
Metagenomics>PCR = BRSV
Overlapping Crl = BPIV-3 and IDV

Specificity: PCR>Metagenomics = none
Metagenomics>PCR = BCoV
Overlapping Crl = BoHV-1, BPIV-3, BRSV, IDV

The 95% CrI overlapped for Se and Sp for all viruses when comparing estimates that did not adjust for the water control data (Table S2a) vs those that did adjust for the mean of virus counts from the water control data and were presented in the main paper (Table 4).

Interpretation:
The overall interpretation of the test comparisons from the BLCM are not sensitive to whether the data were adjusted for the water controls.

Supplement A: Part 2 of the sensitivity analysis examining the impact of different options for adjusting for the results of the water controls on the analysis

Table S1b. Bayesian latent class models using viral data that was adjusted for median viral count data from the water controls.

Models compare qPCR and nanopore metagenomics to estimate the sensitivity and specificity of each diagnostic test for detection of five of six viruses associated with BRD in 760 nasal samples collected from commercial feedlot cattle.

Virus	Test	Sensitivity			Specificity		
		Lower95	Median	Upper95	Lower95	Median	Upper95
**BCoV**	PCR	0.80	**0.89**	0.999	0.51	**0.59**	0.67
	Metagenomics reads ≥ 30	0.26	**0.35**	0.47	0.87	**0.91**	0.94
**BoHV-1**	PCR	0.19	**0.39**	0.99	0.85	**0.93**	0.996
	Metagenomics reads ≥ 1	0.01	**0.04**	0.15	0.99	**0.998**	0.999
**BPIV-3**	PCR	0.21	**0.42**	0.67	0.80	**0.86**	0.93
	Metagenomics reads ≥ 5	0.18	**0.50**	0.86	0.89	**0.94**	0.999
**BRSV**	PCR	0.22	**0.32**	0.43	0.96	**0.98**	0.999
	Metagenomics reads ≥ 1	0.44	**0.60**	0.77	0.89	**0.94**	0.99
**IDV**	PCR	0.53	**0.66**	0.80	0.85	**0.92**	0.999
	Metagenomics reads ≥ 1	0.48	**0.60**	0.72	0.82	**0.90**	0.97

Abbreviations:

BCoV: bovine coronavirus; BoHV-1: bovine herpes virus; BPIV-3: bovine parainfluenzavirus 3; BRSV: bovine respiratory syncytial virus; BVDV: bovine viral diarrhea virus 1 and 2; IDV: influenza D virus; Lower95: lower band of 95% credible interval; Upper95: upper band of 95% credible interval; qPCR: quantitative polymerase chain reaction. Estimates were not available for detection of BVDV (bovine viral diarrhea virus 1 and 2), as there were no qPCR positive test results.

Sensitivity: PCR>Metagenomics = BCoV, BoHV-1
Metagenomics>PCR = BRSV
Overlapping Crl = BPIV-3 and IDV

Specificity PCR>Metagenomics = none
Metagenomics>PCR = BCoV
Overlapping Crl = BoHV-1, BPIV-3, BRSV, IDV

The 95% CrI overlapped for Se and Sp for all viruses when comparing estimates that adjusted for the median viral count data from the water controls (Table S2b) vs those that adjusted for the mean of virus counts from the water control data and were presented in the main paper (Table 4).

Interpretation:
The overall interpretation of the test comparisons from the BLCM are not sensitive to whether the data were adjusted using the mean or median of virus counts for the water controls.

Supplement B: Sensitivity analysis examining the impact of the covariance term in the BLCM model for detection of BPIV-3 in a two test and two population model

Table S2a. Results from the Bayesian latent class model comparing qPCR and metagenomics for detection of BPIV-3 that did not include the covariance term.

* New model constructed to assess the importance of including the covariance term in the model

BPIV-3 ≥ 5, no covariance

Virus	Test	Sensitivity			Specificity		
		Lower95	**Median**	Upper95	Lower95	**Median**	Upper95
BPIV-3	PCR	0.23	**0.37**	0.57	0.81	**0.86**	0.93
	Metagenomics	0.19	**0.39**	0.71	0.89	**0.93**	0.99

Table S2b. Results from the Bayesian latent class model comparing qPCR and metagenomics for detection of BPIV-3 that included the covariance term.

* Model reported in the main text of the analysis for comparison

BPIV-3 ≥ 5, with covariance

Virus	Test	Sensitivity			Specificity		
		Lower95	**Median**	Upper95	Lower95	**Median**	Upper95
BPIV-3	PCR	0.21	**0.42**	0.66	0.80	**0.86**	0.92
	Metagenomics	0.19	**0.52**	0.87	0.89	**0.94**	0.999
	Covariance	-0.14	**-0.02**	0.06	-0.01	**0.00**	0.02

Interpretation:

The 95% CrI for the estimates for the models with and without covariance overlap, suggesting the model was not sensitive to the inclusion of the covariance term.

This assumption was examined for BPIV-3 alone, as it was the only model limited to two populations, resulting in fewer degrees of freedom to estimate covariance parameters.

Supplement C:

Table S3. Kappa statistics measuring agreement beyond chance of metagenomic sequencing vs qPCR or culture for the detection of five viruses and four bacteria of interest in 760 nasal samples collected from commercial feedlot cattle

	Absolute agreement	Kappa*	Standard error	Number of reads for sequencing sample to be positive
**Viral metagenomics vs qPCR**				
**BRSV**	70%	0.12	0.033	≥ 1 read
**IDV**	73%	0.39	0.036	≥ 1 read
**BCoV**	50%	0.11	0.025	≥ 30 reads
**BoHV-1**	84%	0.076	0.015	≥ 1 read
**BPIV-3**	75%	0.11	0.036	≥ 5 reads
**Viral metagenomics vs culture**				
Mannheimia haemolytica	61%	0.15	0.033	≥ 14 reads
Pasteurella multocida	77%	0.30	0.036	≥ 9 reads
Histophilus somni	75%	0.16	0.025	≥ 8 reads
**Viral metagenomics vs qPCR**				
Mycoplasmopsis bovis	81%	0.39	0.036	≥ 1 read
	83%	0.41	0.033	≥ 2 reads**

* These values were not presented in the main paper as kappa is well known to be very sensitive to low prevalence but are included here for completeness and to allow comparison to other publications.

** ≥ 2 reads presented as a sensitivity analysis to the ≥ 1 read presented in the primary paper.

Interpretation of kappa (agreement beyond what is expected by chance): As described by Dohoo, Martin, and Stryhn (1) ($\kappa < 0.00$ – poor, 0.01-0.20 – slight, 0.21-0.40 – fair, 0.41-0.60 – moderate, 0.61-0.80 – substantial, 0.81-1.00 – near perfect).

Reference

1. Dohoo IR, Martin W, Stryhn HE. *Veterinary Epidemiologic Research*. 2nd ed. Charlottetown, P.E.I., Canada: VER Inc. (2014).
