# Supplementary material for: Diagnostic sensitivity and specificity of metagenomic sequencing and qPCR for detection of viruses associated with bovine respiratory disease estimated using Bayesian latent class models
Source: Front Vet Sci. 2026 Feb 25;13:1704414. doi: 10.3389/fvets.2026.1704414 (PMC12976743; doi:10.3389/fvets.2026.1704414)
Supplement: SUPPLEMENTARY MATERIAL 3 — Assessment of BRD bacteria detection by nanopore sequencing (protocol optimized for virus detection), culture and qPCR using Bayesian latent class analysis: Methods and supplemental results. [file Supplementary_file_3.pdf]

Diagnostic sensitivity and specificity of metagenomic sequencing and qPCR for detection of viruses associated with bovine respiratory disease estimated using Bayesian latent class models

Supplementary Materials 3:

Assessment of BRD bacteria detection by nanopore sequencing (protocol optimized for virus detection), culture and qPCR using Bayesian latent class analysis: Methods and supplemental results

Introduction

Bacterial reads of interest were detected from the same metagenomic sequencing dataset used for viral analysis. However, to best interpret these values given potential issues with barcode crosstalk, it was necessary to develop evidence-based recommendations for classifying the results for samples having detected or not detected the bacteria of interest. Another concurrent study generated phenotype data (bacterial culture) from deep nasopharyngeal samples (DNPS) collected at the same time points from the same animals (1). This provided an opportunity to generate read count cutoff thresholds based on receiver operating characteristic ROC curves and then adjusting the cutoffs as necessary to meet minimum specificity criteria based on Bayesian latent class models by comparing the bacterial data to culture for three bacteria of interest: *Mannheimia haemolytica*, *Pasteurella multocida*, and *Histophilus somni*. The DNPS collected from the concurrent study were also tested using a qPCR assay for *Mycoplasmopsis bovis* (formerly called *Mycoplasma bovis*), and a read count cutoff threshold for detection was generated based on a ROC curve by comparing bacterial read counts to qPCR results for *M. bovis*. There was insufficient ARG data generated within the viral metagenomic sequence data to establish ROC-based cutoffs for the detection of potential AMR determinants in these samples.

Objectives

The first objective was to compare bacterial reads from a viral metagenomic sequencing dataset and bacterial culture results to develop ROCs and then BLCMs to estimate sensitivity and specificity for each diagnostic method in the absence of a gold standard for detection of *M. haemolytica*, *P. multocida*, and *H. somni*. The second objective was to compare bacterial reads from a viral metagenomic sequencing dataset and qPCR to develop a BLCM to estimate sensitivity and specificity for each diagnostic method in the absence of a gold standard for detection of *M. bovis*.

Materials and Methods

The ethics statement, animal population and animal sampling procedures were described in the main text of the paper. All laboratory work reported here for culture, DNA extraction and qPCR

was completed by a regional commercial laboratory (Prairie Diagnostic Services Inc., Saskatoon, SK).

Concurrent with collection of the nasal swab for the viral metagenomics and qPCR described in the main paper, additional deep nasal pharyngeal swabs (DNPS) were collected as part of the Canadian Feedlot Antimicrobial Use and Antimicrobial Resistance Surveillance Program (CFAASP) (https://cfaasp.ca/resources/cfaasp-resources/Bovine-Respiratory-Disease-BRD-Pathogen-Antimicrobial-Resistance-AMR-Update-2022). These swabs provided access to culture and *M. bovis* qPCR data for comparison to the data generated by the matching nasal swabs analyzed with viral metagenomics and viral qPCR.

As described in the main paper, registered veterinary technologists with extensive training in feedlot settings utilized commercial cattle chutes and headgates equipped with neck extenders to restrain cattle for the collection of swabs. Two deep nasal pharyngeal swabs (DNPS) were collected from each animal, from a convenience sample of 20 cattle from the 13 pens FPC and the 6 pens YRL at arrival processing before metaphylaxis. DNPS were collected from another convenience sample from the same pens again at approximately 14 DOF, however, the same cattle were not necessarily resampled.

At each sampling time point, calves were restrained in a hydraulic chute and sampled with a neck extender used to stabilize each calf's head during sampling. A single-use paper towel was used to wipe clean the external nares, and a double-guarded culture swab (Continental Plastic Corp., Delevan, WI, USA) was directed into the ventral meatus of the nostril. The polyester-tipped swab was advanced through the inner sheath and vigorously rotated against the nasopharyngeal mucosa for 5–6 rotations. The swab was withdrawn into the inner sheath and outer guard prior to removal from the nostril. Immediately after sample collection, the swab was pushed through the guard into a vial of Amies transport media (Micronostyx, Ottawa, Canada). The excess length of the swab was cut off, and the tube tightly sealed. One additional sample was obtained from the alternate nostril. After completing the required sample collection, samples were couriered in an insulated cooler with ice packs to the diagnostic laboratory at the University of Saskatchewan, Prairie Diagnostic Services Inc. (PDS), Saskatoon, SK for bacterial culture and qPCR for *M. bovis*.

Meta-data were collected using the CFAASP sample submission sheet, including the estimated average weight of calves in the pen and animal age group (calf/yearling). The days-on-feed (DOF) for the second sampling time point was determined using the date relative to the first sample (arrival processing), which was collected at one DOF.

Methods for bacterial culture

The fall 2022 data for bacterial cultures results including these samples have been previous reported by Canadian Feedlot Antimicrobial Use and Antimicrobial Resistance Surveillance Program (CFAASP) (https://cfaasp.ca/resources/cfaasp-resources/Bovine-Respiratory-Disease-BRD-Pathogen-Antimicrobial-Resistance-AMR-Update-2022). The methods used to generate the culture data were briefly summarized.

Bacterial cultures were initiated by inoculating 5% Columbia sheep blood (BA) and Chocolate agar (CHOC) plates with the DNP swab tip. The swab tip was stored at -80° C for subsequent DNA extraction for *M. bovis* PCR.

Plates were incubated at 35 °C for 18 h in 5% CO2. Bacterial colonies were examined at 18 h and 42 h of incubation. By examining both BA and CHOC plates, one isolate exhibiting phenotypic morphologies for each bacterium of interest was selected and confirmed using MALDI-TOF MS (Bruker Daltonik, Bremen, Germany), according to manufacturer guidelines. MALDI-TOF MS Biotyper Microflex LT Compass version 1.4 software and the MSP library were used for direct testing. If visible characteristics suggested the presence of multiple species of interest from one sample, representative colonies of each unique colony morphology were selected for identification. Positive and negative controls were processed for each day of sample setup and for each new media lot using Staphylococcus aureus ATCC 29213, Escherichia coli ATCC 25922, and Histophilus somni ATCC 700025. Only MALDI-TOF identification scores of ≥2 indicating secure species-level identification were used for further analysis. A plain matrix spot was run with every MALDI run to ensure no contamination.

M. bovis **qPCR testing**.

Following bacterial culture, DNA extraction was completed on the remaining volume from the raw sample using the MagMAX™ CORE Nucleic Acid Purification Kit (Thermo Fisher Scientific) according to manufacturer guidelines, and the KingFisher™ Flex Purification System,

KingFisher with 96 PCR head (Thermo Fisher Scientific). The process yielded an approximate final elution volume of 100 µL of extracted DNA from each sample, which was stored at 4 °C until testing.

The number of *M. bovis* uvrC target gene copies were quantified by qPCR on a BioRad CFX96 Real Time PCR Detection System (BIO-RAD, Hercules, California, United States). Enzyme activation was completed for two minutes at 95 °C, followed by 40 cycles of amplification, consisting of five seconds at 95 °C and 33 seconds at 60° C. Samples were considered 'negative' if no amplification was detected, 'suspect' for *M. bovis* at a cycle threshold (Ct) value of 37.0 to 40.0, and 'positive' for the presence of *M. bovis* at a Ct value of less than 37.0.

Statistical Analysis

Data management and statistical analyses

Animal data, culture and PCR results together with the bacterial metagenomic data generated by the viral protocol described in the main manuscript were managed in a spreadsheet (Microsoft Excel, version 2401, Microsoft Corporation, Redmond, Washington, DC, USA), and descriptive analyses were completed using a commercial statistical software package (Stata/IC, version 16.1, StataCorp LLC, College Station, TX, USA).

Bayesian Latent Class Models

Bayesian latent class models were developed and run using JAGS software (2) and the runjags package (3) in R (R Foundation for Statistical Computing, Vienna, Austria). Uninformative priors (beta(1,1)) were used for sensitivity, specificity, and prevalence for each population. Convergence was evaluated using diagnostics including potential scale reduction factor (<1.05), effective sample size (>1000), and Monte Carlo standard errors as a percent of standard deviation (<5%) as well as visual inspection of trace and autocorrelation plots and deemed satisfactory for all models. Results were reported as medians of the posterior distributions along with 95% credible intervals (CrIs). Two-test (culture (or PCR) vs viral long-read metagenomic sequencing protocol), four-population BLCMs were used for the analysis of the bacterial data. The four populations considered were the fall-placed calves and yearlings at arrival processing and again at 14 DOF as described in the main manuscript.

Bayesian latent class models were developed to compare the diagnostic test performance of culture and the viral metagenomic sequencing protocol for the detection of *M. haemolytica, P. multocida*, and *H. somni*, and qPCR and the viral metagenomic sequencing protocol for the detection of *M. bovis*. The latent class for these models was defined as the presence of the organism in DNP samples. The classification of a sample as positive or negative by metagenomic sequencing was estimated using two metrics: first, total number of reads identified as was done for the virus reads and second, theoretical coverage as was used to capture the additional value associated by accounting for the variation in read lengths and total read length captured by reads reported for each sample for *M. haemolytica, P. multocida* and *H. somni*. Theoretical coverage for each sample was calculated by dividing the total read length for each of the bacteria of interest divided by the size of its reference genome (*M. haemolytica*: 2.8 Mb [NCBI GCF_002285575.1], *P. multocida*: 2.3 Mb [NCBI CF_002073255.2], *H. somni*: 2.3 Mb [NCBI GCF_000019405.1]).

The number of identified reads for each organism and then the theoretical coverage were each evaluated against culture results using receiver operating characteristic (ROC) curves in R (pROC, (4)) to determine a baseline cutoff based on maximizing combined sensitivity and specificity (Youden's index) of metagenomics compared to culture or PCR in the case of *M. bovis*. This baseline cutoff was used for an initial classification of samples as positive or negative for comparison to culture/PCR results using BLCM (Figure S1).

Results were compared for both read count and theoretical coverage across all bacteria of interest. To be consistent with what is reported for the virus work, read counts corrected for the average of the water controls for each run was selected for further analyses to optimize cutoff values. If the cutoff resulting from the initial ROC analysis optimizing the best balance of sensitivity and specificity resulted in a specificity of at least 0.90 for metagenomics, the cutoff was considered adequate (Figure S1). If the initial BLCM resulted in a specificity of < 0.90, the cutoff was increased by increments of 1 read and the BLCM repeated until a specificity of ≥ 0.90 for metagenomics was obtained. The resultant cutoff was then used to determine the classification of metagenomics sequencing as positive or negative for the final models reported (Figure S1). The Bayesian latent class models for bacteria assume conditional independence between diagnostic tests with the exception of the *M. bovis* model.

To explore the impact of different approaches for adjusting read counts to account for contamination/barcode crosstalk based on read counts for each bacteria in the water controls, the initial BLCM analyses were repeated without adjustment for the water controls, and after adjusting for the median read counts (rather than average) for each bacteria in the water controls, and are reported in this Appendix.

Figure S1. Summary of the workflow for determining thresholds for identifying the samples containing target bacteria based on bacterial read counts as informed by Bayesian latent class models.

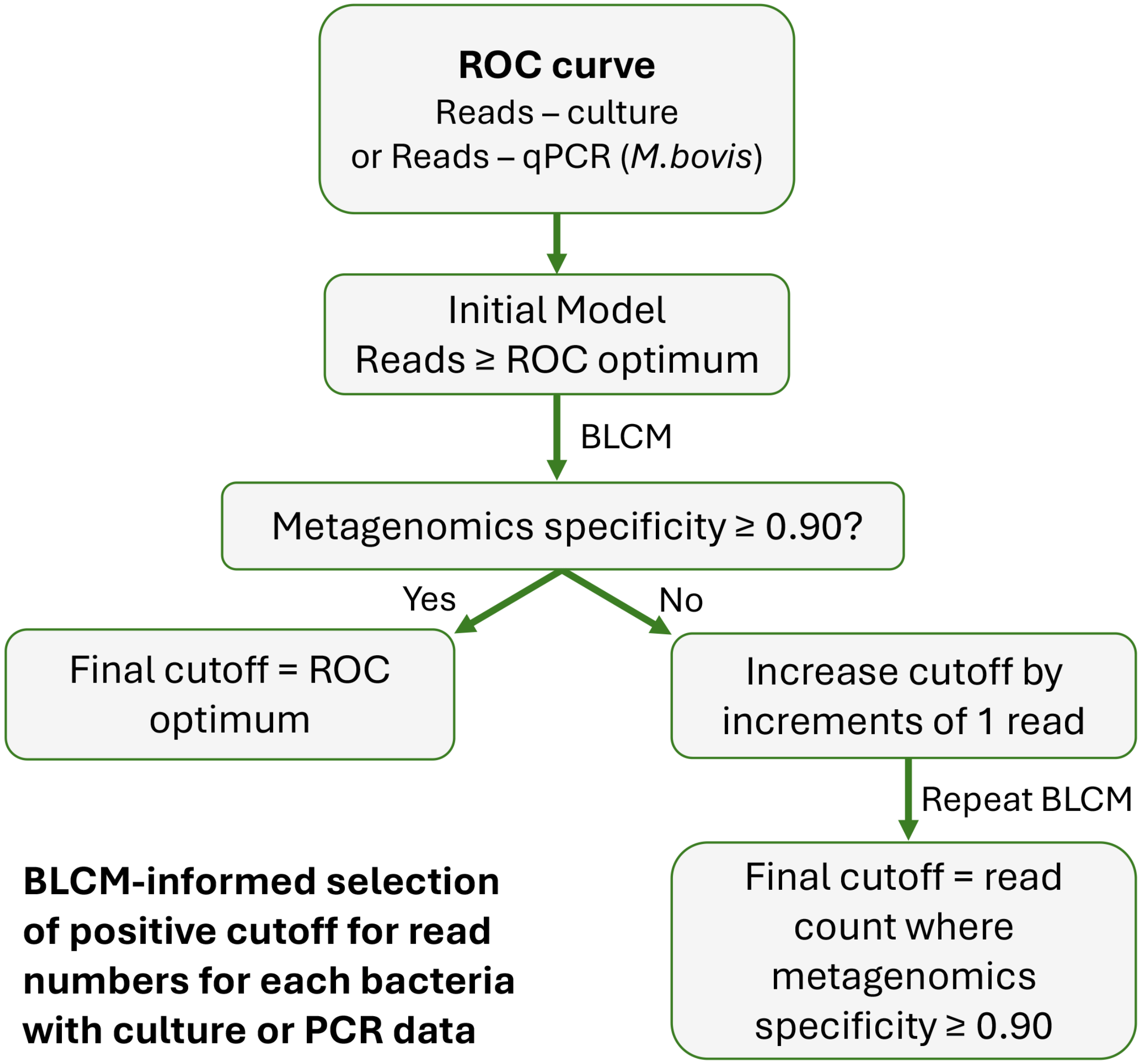

Abbreviations: BLCM: Bayesian latent class model; qPCR: quantitative polymerase chain reaction; ROC =: receiver operating characteristic=.

Results

Detailed descriptions of the bacterial metagenomics results were reported in a companion paper (5). A summary of the different cutoffs identified by the ROC curves for both read counts and theoretical coverage are presented in Table S1 below with the associated summary results for the BLCM. Results are presented for the raw data, the data corrected for the mean of the three water controls per flow cell, and the data corrected for the median of the three water controls per flow cell. The detailed results for each model including 95%CrI are presented in Table S2 for read numbers and Table S3 for theoretical coverage for *M. haemolytica*, *P. multocida* and *H. somni*. The results for *M. bovis* are summarized in Table S4.

The final model results with the read count cutoffs to meet a threshold ≥0.90 for specificity for four bacteria of interest were summarized in the main paper in Table 9.

Table S1. Compare Bayesian Latent Class Model results for classifications based on ROC curve-derived thresholds for read numbers and theoretical coverage across different adjustment options for water controls.

		Mannheimia haemolytica					*Pasteurella multocida*				
		**cutoff (ROC)**	**positive count**	**kappa**	**Se**	**Sp**	**cutoff (ROC)**	**positive count**	**kappa**	**Se**	**Sp**
**Read count**	**raw**	20.5	305	0.15	0.67	0.88	8.5	174	0.29	0.51	0.87
	**mean WC**	8.5	364	0.15	0.73	0.85	4.5	193	0.29	0.57	0.83
	**median WC**	14.5	324	0.15	0.64	0.91	2.5	259	0.25	0.65	0.75
**Theoretical coverage**	**raw**	0.0005	335	0.12	0.64	0.78	0.0006	218	0.21	0.51	0.77
	**mean WC**	0.0005	297	0.15	0.64	0.86	0.0002	255	0.21	0.59	0.73
	**median WC**	0.0005	302	0.14	0.59	0.85	0.0001	311	0.19	0.67	0.67
**culture**			183					177			

		Histophilus somni					*Mycoplasmopsis bovis*				
		**cutoff (ROC)**	**positive count**	**kappa**	**Se**	**Sp**	**cutoff (ROC)**	**count**	**kappa**	**Se**	**Sp**
**Read count**	**raw**	7.5	220	0.16	0.73	0.91	>=1	174	0.43	0.69	0.98
	**mean WC**	7.5	210	0.16	0.71	0.92	>=1	123	0.39	0.45	0.98
	**median WC**	7.5	218	0.16	0.73	0.92	>=1	126	0.36	0.46	0.98
**Theoretical coverage**	**raw**	0.0006	94	0.27	0.55	0.96					
	**mean WC**	0.0005	112	0.24	0.57	0.94					
	**median WC**	0.0005	117	0.23	0.57	0.94					
**culture**			42				**PCR** < 37.0 CT	174			

Table S2. Results from Bayesian Latent Class Models based on thresholds from ROC curves bacteria data generated from virus pipeline (read counts).

	M. haemolytica			*P. multocida*			*H. somni*		
				**Reads, raw data**					
	L95	**Median**	U95	L95	**Median**	U95	L95	**Median**	U95
se[1] - culture	0.27	**0.35**	0.45	0.52	**0.75**	1.00	0.11	**0.17**	0.25
se[2] - metagenomics	0.50	**0.67**	0.92	0.38	**0.51**	0.69	0.57	**0.73**	0.89
sp[1] - culture	0.79	**0.87**	0.97	0.88	**0.94**	1.00	0.98	**1.00**	1.00
sp[2] - metagenomics	0.76	**0.88**	1.00	0.81	**0.87**	0.94	0.85	**0.91**	0.98
prev[1] - T1 calves	0.17	**0.39**	0.61	0.25	**0.41**	0.60	0.27	**0.42**	0.57
prev[2] - T1 yearlings	0.04	**0.30**	0.57	0.02	**0.19**	0.40	0.00	**0.06**	0.15
prev[3] - T2 calves	0.39	**0.66**	0.92	0.05	**0.15**	0.28	0.29	**0.43**	0.57
prev[4] - T2 yearlings	0.29	**0.61**	0.91	0.05	**0.18**	0.40	0.00	**0.09**	0.20
				**Reads, mean WC corrected**					
	L95	**Median**	U95	L95	**Median**	U95	L95	**Median**	U95
se[1] - culture	0.27	**0.34**	0.43	0.62	**0.83**	1.00	0.11	**0.17**	0.25
se[2] - metagenomics	0.59	**0.73**	0.93	0.43	**0.57**	0.74	0.54	**0.71**	0.87
sp[1] - culture	0.81	**0.89**	0.98	0.87	**0.93**	1.00	0.98	**1.00**	1.00
sp[2] - metagenomics	0.70	**0.85**	1.00	0.79	**0.83**	0.88	0.86	**0.92**	0.99
prev[1] - T1 calves	0.21	**0.43**	0.63	0.23	**0.37**	0.52	0.28	**0.43**	0.58
prev[2] - T1 yearlings	0.11	**0.39**	0.66	0.03	**0.16**	0.32	0.00	**0.06**	0.15
prev[3] - T2 calves	0.44	**0.69**	0.91	0.05	**0.13**	0.24	0.28	**0.42**	0.57
prev[4] - T2 yearlings	0.49	**0.75**	0.98	0.02	**0.12**	0.25	0.00	**0.09**	0.20
				**Reads, median WC corrected**					
	L95	**Median**	U95	L95	**Median**	U95	L95	**Median**	U95
se[1] - culture	0.27	**0.34**	0.42	0.57	**0.77**	1.00	0.11	**0.17**	0.24
se[2] - metagenomics	0.51	**0.64**	0.84	0.51	**0.65**	0.81	0.57	**0.73**	0.89
sp[1] - culture	0.83	**0.91**	1.00	0.87	**0.93**	1.00	0.98	**1.00**	1.00
sp[2] - metagenomics	0.78	**0.91**	1.00	0.70	**0.75**	0.81	0.85	**0.92**	0.99
prev[1] - T1 calves	0.25	**0.46**	0.64	0.25	**0.40**	0.58	0.28	**0.43**	0.58
prev[2] - T1 yearlings	0.18	**0.45**	0.68	0.02	**0.17**	0.34	0.00	**0.06**	0.15
prev[3] - T2 calves	0.52	**0.77**	0.97	0.06	**0.16**	0.28	0.30	**0.43**	0.57
prev[4] - T2 yearlings	0.42	**0.72**	0.96	0.01	**0.13**	0.27	0.00	**0.10**	0.21

*** WC - water control**

Table S3. Results from Bayesian Latent Class Models based on thresholds from ROC curves bacteria data generated from virus pipeline (theoretical coverage).

	M. haemolytica			***P. multocida***			***H. somni***		
				**Theoretical coverage, raw data**					
	L95	**Median**	U95	L95	**Median**	U95	L95	**Median**	U95
se[1] - culture	0.26	**0.35**	0.49	0.66	**0.88**	1.00	0.17	**0.30**	0.46
se[2] - metagenomics	0.50	**0.64**	0.89	0.39	**0.51**	0.66	0.36	**0.55**	0.80
sp[1] - culture	0.79	**0.87**	0.98	0.87	**0.94**	1.00	0.98	**0.99**	1.00
sp[2] - metagenomics	0.63	**0.78**	0.97	0.73	**0.77**	0.81	0.93	**0.96**	0.99
prev[1] - T1 calves	0.15	**0.43**	0.69	0.23	**0.35**	0.50	0.05	**0.15**	0.26
prev[2] - T1 yearlings	0.02	**0.34**	0.64	0.02	**0.16**	0.31	0.00	**0.04**	0.11
prev[3] - T2 calves	0.31	**0.63**	0.92	0.03	**0.14**	0.24	0.17	**0.30**	0.46
prev[4] - T2 yearlings	0.30	**0.66**	1.00	0.03	**0.13**	0.25	0.00	**0.03**	0.12
				**Theoretical coverage, mean WC corrected**					
	L95	**Median**	U95	L95	**Median**	U95	L95	**Median**	U95
se[1] - culture	0.28	**0.36**	0.47	0.62	**0.85**	1.00	0.15	**0.29**	0.46
se[2] - metagenomics	0.48	**0.64**	0.89	0.46	**0.59**	0.76	0.37	**0.57**	0.78
sp[1] - culture	0.80	**0.88**	0.98	0.87	**0.93**	1.00	0.98	**0.99**	1.00
sp[2] - metagenomics	0.74	**0.86**	1.00	0.69	**0.73**	0.78	0.90	**0.94**	0.99
prev[1] - T1 calves	0.16	**0.39**	0.61	0.22	**0.36**	0.53	0.06	**0.17**	0.29
prev[2] - T1 yearlings	0.04	**0.33**	0.60	0.00	**0.14**	0.30	0.00	**0.04**	0.11
prev[3] - T2 calves	0.34	**0.62**	0.86	0.05	**0.15**	0.27	0.17	**0.31**	0.47
prev[4] - T2 yearlings	0.36	**0.68**	0.98	0.02	**0.12**	0.24	0.00	**0.05**	0.15
				**Theoretical coverage, median WC corrected**					
	L95	**Median**	U95	L95	**Median**	U95	L95	**Median**	U95
se[1] - culture	0.27	**0.36**	0.48	0.54	**0.76**	1.00	0.15	**0.27**	0.43
se[2] - metagenomics	0.47	**0.59**	0.80	0.54	**0.67**	0.84	0.38	**0.57**	0.79
sp[1] - culture	0.82	**0.91**	1.00	0.86	**0.92**	1.00	0.98	**0.99**	1.00
sp[2] - metagenomics	0.72	**0.85**	1.00	0.61	**0.67**	0.73	0.90	**0.94**	0.99
prev[1] - T1 calves	0.20	**0.45**	0.67	0.23	**0.39**	0.60	0.06	**0.17**	0.30
prev[2] - T1 yearlings	0.10	**0.41**	0.68	0.00	**0.14**	0.32	0.00	**0.04**	0.11
prev[3] - T2 calves	0.34	**0.64**	0.87	0.05	**0.16**	0.30	0.18	**0.33**	0.50
prev[4] - T2 yearlings	0.44	**0.77**	1.00	0.00	**0.11**	0.25	0.00	**0.05**	0.17

*** WC - water control**

Table S4. Results for Bayesian Latent Class Models for *Mycoplasmopsis bovis* bacteria data generated from virus pipeline (read counts).

		M. bovis	
Raw read counts ≥ 1	L95	**Median**	U95
se[1] - qPCR	0.43	0.56	0.68
se[2] – viral metagenomics	0.52	0.69	0.85
sp[1] - qPCR	0.89	0.92	0.95
sp[2] – viral metagenomics	0.96	0.98	1.00
prev[1] - T1 calves	0.00	0.01	0.04
prev[2] - T1 yearlings	0.00	0.05	0.13
prev[3] - T2 calves	0.63	0.78	1.00
prev[4] - T2 yearlings	0.10	0.23	0.39
covse12	-0.06	0.02	0.08
covsp12	0.00	0.00	0.01
Mean water control corrected ≥ 1	L95	**Median**	U95
se[1] - qPCR	0.44	0.61	0.81
se[2] – viral metagenomics	0.31	0.45	0.61
sp[1] - qPCR	0.92	0.96	0.99
sp[2] – viral metagenomics	0.96	0.98	1.00
prev[1] - T1 calves	0.00	0.02	0.06
prev[2] - T1 yearlings	0.01	0.13	0.26
prev[3] - T2 calves	0.53	0.72	0.99
prev[4] - T2 yearlings	0.21	0.38	0.60
covse12	-0.08	0.02	0.08
covsp12	0.00	0.00	0.01
Mean water control corrected ≥ 1	L95	**Median**	U95
se[1] - qPCR	0.43	0.60	0.78
se[2] – viral metagenomics	0.32	0.46	0.61
sp[1] - qPCR	0.92	0.96	0.99
sp[2] – viral metagenomics	0.96	0.98	1.00
prev[1] - T1 calves	0.00	0.02	0.06
prev[2] - T1 yearlings	0.01	0.13	0.25
prev[3] - T2 calves	0.55	0.73	1.00
prev[4] - T2 yearlings	0.21	0.38	0.59
covse12	-0.09	0.00	0.07
covsp12	0.00	0.00	0.01

References

1. Canadian Feedlot Antimicrobial Use and Antimicrobial Resistance Surveillance Program (CFAASP). Bovine Respiratory Disease (BRD) Pathogen Antimicrobial Resistance (AMR) Update 2022 (2023) [Aug 14, 2025]. Available from: https://cfaasp.ca/resources/cfaasp-resources/Bovine-Respiratory-Disease-BRD-Pathogen-Antimicrobial-Resistance-AMR-Update-2022.
2. Plummer M, editor. JAGS: A program for analysis of Bayesian graphical models using Gibbs sampling. *3rd International Workshop on Distributed Statistical Computing*; 2003; Vienna, Austria.
3. Denwood MJ. Runjags: An R package providing interface utilities, model templates, parallel computing methods and additional distributions for MCMC models in JAGS. *Journal of Statistical Software.* (2016) 71(9):1 - 25. doi: 10.18637/jss.v071.i09.
4. Robin X, Turck N, Hainard A, Tiberti N, Lisacek F, Sanchez JC, et al. pROC: an open-source package for R and S+ to analyze and compare ROC curves. *BMC Bioinformatics.* (2011) 12:77. doi: 10.1186/1471-2105-12-77.
5. Donbraye E, McLeod L, Carson CN, Chai Z, Lacoste SR, Herman EK, et al. Prevalences of respiratory viruses and bacteria in Western Canadian commercial feedlot calves detected using a single metagenomic sequencing protocol vary during the first two weeks of arrival and by age group. *Front Vet Sci.* (2025) 12:1704412. doi: 10.3389/fvets.2025.1704412.
